# Investigating the Epigenetic Therapeutic Potential of Natural Compounds in Cancer

**DOI:** 10.3390/ijms262110776

**Published:** 2025-11-05

**Authors:** Agnieszka Zaczek, Aleksandra Rodacka

**Affiliations:** Department of Oncobiology and Epigenetics, Faculty of Biology and Environmental Protection, University of Lodz, 141/143 Pomorska Street, 90-236 Lodz, Poland

**Keywords:** epigenetics modifications, natural compounds, cancer progression

## Abstract

Natural compounds, including alkaloids, terpenes, and polyphenols, are increasingly recognized for their potential to modulate epigenetic mechanisms and influence cellular function, particularly in cancer. Studies have shown that diverse phytochemicals impact epigenetic modifications, such as DNA methylation, histone modifications, and non-coding RNA expression. Epigenetics is critical in cancer and can dysregulate crucial cellular processes, such as cell proliferation, apoptosis, and differentiation. In cancer, aberrant epigenetic patterns can silence tumor suppressor genes and activate oncogenes, contributing to uncontrolled cell growth and metastasis. Understanding the precise mechanisms by which these natural compounds interact with the epigenetic machinery holds significant promise for developing novel therapeutic strategies for cancer and other diseases. Future research, including basic studies and well-designed clinical trials, will be crucial in validating these findings and developing effective clinical applications of natural compounds.

## 1. Introduction

Epigenetic modulation, defined as the alteration of gene expression patterns without concomitant changes in DNA sequence, provides a dynamic interface through which environmental exposures, including dietary constituents, can influence cellular phenotypes, particularly in neoplastic transformation. The epigenetic modifications, such as DNA methylation, histone post-translational modifications, and non-coding RNA expression, are inherently reversible, presenting opportunities for both chemopreventive and therapeutic interventions [1].

Changes in epigenetic modifications in cancer cells include the regulation of suppressor genes, oncogenes, genes involved in proliferation, migration, invasion, and angiogenesis. Recent research has shown that natural compounds play a significant role in modulating cancer progression by regulating epigenetic mechanisms. This observation has stimulated investigations into the potential of dietary compounds to reverse these aberrant modifications and restore homeostatic gene expression. This review critically examines the epigenetic effects of alkaloids, terpenes, and polyphenols and their implications for cancer therapeutics. These phytochemicals, prevalent in plant-based diets, exhibit the capacity to modulate epigenetic mechanisms through diverse molecular pathways, encompassing the regulation of DNA methyltransferases, histone-modifying enzymes, and microRNA [2,3].

A comprehensive understanding of the precise molecular mechanisms by which these dietary compounds interact with the epigenetic machinery is essential for the development of novel, epigenetically targeted cancer therapies as well as preventive therapies. This introduction lays the groundwork for a detailed analysis of the current state of knowledge regarding selected dietary compound-mediated epigenetic modulation and its potential to translate into clinically relevant anticancer strategies.

## 2. Epigenetic Effects of Natural Compounds: Alkaloids, Terpenes, and Polyphenols

### 2.1. Alkaloids

Alkaloids are a vast class of naturally occurring organic compounds primarily found in plants. Alkaloids can be found in various organisms, but plants are by far the most prolific producers. Families such as the *Leguminosae* (beans, peas, lentils), *Menispermaceae* (moonseeds), *Ranunculaceae* (buttercups), *Loganiaceae* (strychnine tree), and *Papaveraceae* (poppies) are particularly rich sources. The specific alkaloids produced by a plant can vary widely, depending on factors like species, geographical location, and growth conditions [4]. Alkaloids have long captivated scientists due to their diverse biological activities. Among these, their potential as anticancer agents has been a subject of extensive research. Many alkaloids disrupt the process of cell division, preventing cancer cells from multiplying. Alkaloids can cause damage to the DNA and trigger programmed cell death in cancer cells, a natural mechanism to eliminate damaged or unwanted cells [5]. Alkaloids can suppress angiogenesis, proliferation, migration, and invasion, which are essential for tumor progression. Due to their potent biological activities, alkaloids have garnered significant attention in the realm of cancer therapy [6]. Compounds like camptothecin, vincristine, vinblastine, berberine, sanguinarine, evodiamine, piperine, matrine, and tetrandrine are notable examples of alkaloids that have demonstrated promising anticancer properties. These compounds have the potential to act synergistically with conventional chemotherapeutic agents, enhancing overall treatment efficacy. Encouragingly, several alkaloids are currently undergoing clinical trials, and initial findings suggest a favorable safety profile. This combination of potent anticancer activity and minimal side effects underscores the potential of alkaloids as a valuable asset in the development of novel cancer treatments [7,8].

#### 2.1.1. 3,3′-Diindolylmethane (DIM)

3,3′-Diindolylmethane (DIM) is a compound formed in the body from indole-3-carbinol, a substance found in cruciferous vegetables such as broccoli, cabbage, and cauliflower. DIM has been shown to have anticancer effects in several in vitro and in vivo models, including prostate, breast, and cervical cancers. For example, Wu et al. [9] showed that DIM treatment significantly decreased the methylation status of five CpGs on the nuclear factor-erythroid factor 2-related factor 2 (Nrf2) promoter region in prostate TRAMP-C1 cells in a dose-dependent manner. Additionally, DIM suppressed the protein levels of DNA demethylases (DNMT1, DNMT3b) and histone deacetylases (HDAC2, HDAC3) in these cells. Furthermore, in the TRAMP mouse model, DIM-treated mice demonstrated an overall lower incidence of tumors and Prostatic Intraepithelial Neoplasia (PIN) lesions compared to the control group, suggesting that DIM suppresses the formation and progression of prostate cancer tumors [9].

**Figure 1:**
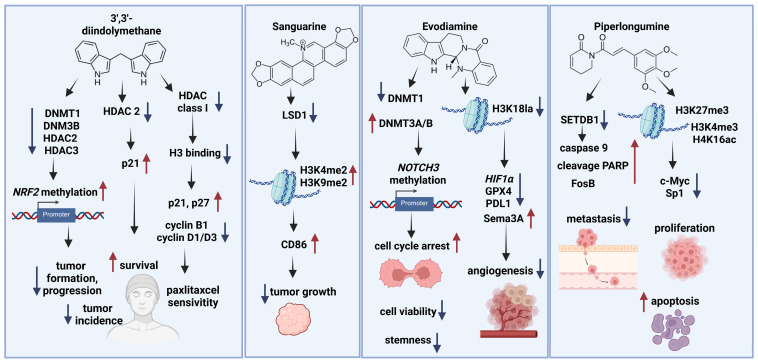
***Epigenetic effects of alkaloids on DNA methylation, histone modification, and associated regulatory enzymes—PART 1.*** Schematic illustration of the effects of alkaloids—3,3′-diindolylmethane, sanguinarine, evodiamine, and piperlongumine—on epigenetic enzymes and histone modifications involved in cancer progression. Each compound modulates specific epigenetic regulators (DNMTs, HDACs, LSD1, SETDB1) and histone marks (e.g., H3K27me3, H3K4me2, H4K16ac, H3K18la), leading to altered expression of genes associated with cell proliferation, apoptosis, metastasis, and survival. **Legend: (↑)** red arrow indicates upregulation, and **(↓)** blue arrow indicates downregulation.

A significant, dose-dependent decrease in HDAC activity was observed in both androgen-dependent (LNCaP) and androgen-independent (PC-3) prostate cancer cells. DIM inhibits HDAC2 activity, which may contribute to the DIM-induced increase in p21 expression in prostate cancer cells. Furthermore, DIM downregulates HDAC2, which is associated with decreased patient survival [10]. In colon cancer cell line HT-29, DIM induced the expression of markers associated with cellular senescence. Lie et al. [11] demonstrated that DIM treatment can deplete class I HDACs, leading to senescence in colon cancer cells. Additionally, DIM decreased histone H3 binding to the p21 and p27 promoters. Furthermore, DIM significantly reduced the levels of cyclins B1, D1, and D3, inducing G2 phase cell cycle arrest in HT-29 cells. The combination of DIM and inhibitor histone deacetylases—suberoylanilide hydroxamic acid (SAHA) resulted in a more pronounced G2 phase arrest compared to DIM alone, suggesting a synergistic interaction between both agents [11] (Figure 1).

DIM may also help overcome resistance to paclitaxel chemotherapy, a major challenge in breast cancer treatment. Paclitaxel (PTX) is a widely used chemotherapeutic agent, but its clinical efficacy is often limited by drug resistance. Studies have suggested that the expression of Krüppel-like factor 4 (KLF4) and DNMT1 may contribute to PTX resistance. Xiang et al. [12] reported that DIM enhanced PTX sensitivity in MCF-7 and T48D breast cancer cells. Mechanistically, DIM effectively decreased DNMT1 expression and reduced the methylation level of the KLF4 promoter, leading to increased KLF4 expression [12]. DIM also induced cell cycle arrest at the G1 or G2 phase by regulating cyclin-dependent kinases and inhibitor kinases dependent on cyclins. Jin et al. [13] showed that DIM’s regulation of cell division cycle 25A (Cdc25A), a key cell cycle regulator, is dependent on its upregulation of miR-21 in MCF-7 cells, but not in ER-negative, p53 mutant MDA-MB-468 cells. miR-21 targets the 3′-UTR of Cdc25A mRNA, leading to reduced Cdc25A expression and subsequent cell cycle arrest [13].

Pancreatic cancer is frequently associated with a highly upregulated expression of miR-221. Experimental evidence indicates that DIM treatment can downregulate miR-221 expression in MiaPaCa-2 and Panc-1 pancreatic cancer cell lines. This downregulation promotes the activation of tumor suppressor genes, including phosphatase and tensin homolog (PTEN), p27, p57, and p53, and upregulates modulator of apoptosis (PUMA), leading to reduced cell proliferation and migration. Moreover, DIM also increased the expression of miR-146 and decreased the levels of epidermal growth factor receptor (EGFR) and nuclear factor-κB (NF-κB), hindering the invasive potential of pancreatic cancer cells [14] (Table 1).

#### 2.1.2. Sanguinairne

Sanguinarine, a plant alkaloid with the chemical name 13-methyl[1,3]benzodioxolo[5,6-c]-1,3-dioxolo[4,5-i]phenanthridium, is derived from the roots of *Sanguinaria canadensis*, *Argemone mexicana*, and other poppy fumaria species [15]. This compound exhibits a wide range of biological activities, including antimicrobial, antioxidant, anti-inflammatory, and proapoptotic properties [16,17,18]. Studies have shown that sanguinarine can effectively block proliferation and induce apoptosis in various types of malignant cells [17,19]. Further biochemical analysis has revealed that sanguinarine acts as a reversible inhibitor of lysine-specific demethylase 1 (LSD1). By inducing the accumulation of histone modifications H3K4me2 and H3K9me2, as well as upregulating cluster of differentiation 86 (CD86), sanguinarine suppresses lung cancer cell growth [20] (Figure 1).

Additionally, sanguinarine can also regulate miR-497-5p in hepatocellular carcinoma cells. In hepatocellular carcinoma HepG2 cells, it enhances the expression of miR-497-5p, which can target the 3′-UTR of the cyclin-dependent kinase 4 (CDK4) mRNA, leading to decreased levels of this kinase and increased cell proliferation [21] (Table 1).

#### 2.1.3. Evodiamine

Evodiamine (EVO), a bioactive compound isolated from the fruit of *Evodia rutaecarpa* (a plant known as Wu-Zhu-Yu in traditional Chinese medicine), exhibits a broad spectrum of biological activities, including anti-inflammatory, anti-obesity, neuroprotective, and anticancer properties [22,23,24,25,26]. Numerous studies have demonstrated that EVO exerts cytotoxic effects on various cancer cell lines by inhibiting cell proliferation, inducing apoptosis, suppressing cell migration and invasion, and increasing the production of reactive oxygen species [27,28,29,30]. In lung cancer, EVO suppresses tumor growth and metastasis, potentially by inhibiting the Notch homolog 3 (NOTCH3) signaling pathway. It upregulates the expression of DNMT3A/3B while downregulating DNMT1 in non-small cell lung cancer cells. The activation of DNMTs, leading to the methylation of the *NOTCH3* promoter, which may contribute to cell cycle arrest, reduced cell viability, and decreased stemness [31].

Beyond DNA methylation, EVO also modulates histone modifications to exert its anticancer effects. It inhibits histone lactylation at the hypoxia-inducible factor 1 alpha (*HIF1A*) promoter, decreasing HIF1A expression, enhancing semaphorin 3A (Sema3A) transcription, and inhibiting programmed cell death ligand 1 (PD-L1). In vivo, evodiamine markedly inhibited the growth of prostate cancer (PCa) xenografts in nude mice. This was accompanied by a significant decrease in the expression of HIF1α, H3K18la, glutathione peroxidase 4 (GPX4), and PD-L1, while simultaneously increasing the expression of Sema3A, a potent anti-angiogenic factor [32] (Figure 1).

EVO also influences microRNA expression, particularly in colorectal cancer miR-429, frequently overexpressed in multiple cancer types, promotes tumorigenesis by targeting tumor suppressor genes and enhancing cell proliferation, migration, and invasion. Evodiamine downregulates the expression of miR-429 in colorectal cancer cells, leading to decreased proliferation and migration through the regulation of *E-cadherin* and partitioning defective 3 (*Par3*). Moreover, evodiamine can induce apoptosis by increasing cleaved caspase-3 and Poly (ADP-ribose) polymerase proteins (PARP) and caspase-3 activity in colorectal cancer cells, potentially through a mechanism involving miR-429 regulation [33].

In ovarian cancer, EVO exerts its anti-tumor effects through the regulation of the NEAT1-miR-152-3p-CDK19 axis. This compound reduces the expression of the long non-coding RNA nuclear enriched abundant transcript 1 (NEAT1). This, in turn, leads to increased levels of the tumor-suppressor microRNA miR-152-3p. By upregulating miR-152-3p, the compound inhibits the expression of cell cycle regulator cyclin-dependent kinase 19 (CDK19). This ultimately results in decreased cell proliferation and increased apoptosis in ovarian cancer cells [34] (Table 1).

#### 2.1.4. Piperlongumine

Piperlongumine (PL), a natural alkaloid derived from long pepper, exhibits selective cytotoxicity towards cancer cells by promoting the accumulation of reactive oxygen species. Recent research has delved into the molecular mechanisms underlying PL’s anticancer effects, particularly focusing on the role of epigenetic regulators. One key finding is the downregulation of histone methyltransferase SETDB1 upon PL treatment in MCF-7 breast cancer cells. Decreased SETDB1 levels lead to increased caspase-9-mediated apoptosis, as evidenced by the cleavage of PARP [35]. Interestingly, reactive oxygen species (ROS) generation by PL is essential for SETDB1 downregulation. Treatment with the antioxidant N-acetyl cysteine (NAC) can partially counteract this effect, indicating that ROS-mediated oxidative stress is involved in the regulation of SETDB1 expression. This suggests an antagonistic interaction between PL and NAC, where the antioxidant attenuates the PL-induced suppression of SETDB1 [35] (Figure 1).

Furthermore, decreased SETDB1 expression is associated with the activation of Fos binding protein (FosB), a transcription factor implicated in cell proliferation and apoptosis. Overexpression of FosB enhances PL-induced cell death, while silencing FosB reduces cell death, suggesting that FosB plays a pro-apoptotic role in PL-treated MCF-7 cells [35] (Figure 1).

Piperlongumine also exerts its anticancer effects through microRNA regulation in lung cancer cells. One such mechanism involves the upregulation of miR-34b-3p, a tumor-suppressor microRNA that targets and inhibits the expression of oncogenes. PL treatment leads to increased expression of miR-34b-3p, which in turn targets and inhibits the expression of transforming growth factor beta receptor 1 (*TGFBR1*). By downregulating *TGFBR1*, miR-34b-3p can suppress cell growth, migration, and invasion, leading to increased cell death in lung cancer cells A549 and H1299 [36].

In osteosarcoma, PL modulates the Janus kinase/signal transducer and activator of transcription 3 (JAK/STAT3) signaling pathway via miR-30d-5p. Hu et al. [37] found that miR-30d-5p directly targets suppressor of cytokine signaling 3 (*SOCS3*), a negative regulator of the JAK/STAT3 signaling pathway. Overexpression of miR-30d-5p in osteosarcoma cells (OS) led to decreased SOCS3 expression and reduced the anti-tumor effects of PL. PL was shown to inhibit miR-30d-5p expression, leading to increased SOCS3 levels and subsequent inhibition of the JAK/STAT3 pathway. This, in turn, affected OS cell proliferation, apoptosis, and metastasis [37].

Piperlongumine rapidly decreased cellular Myelocytomatosis oncogene (c-Myc) expression in pancreatic cancer cells (Panc1), a process associated with ROS-dependent downregulation of miR-27a and miR-17/20a and upregulation of the transcription factors: zinc finger and BTB domain containing 10/4 (ZBTB10, ZBTB4). Piperlongumine decreased the interaction of RNA polymerase II (pol II) with the GC-rich c-Myc promoter, slightly increased the repressive histone mark H3K27me3, and decreased the activating histone marks H3K4me3 and H4K16ac. A similar pattern was observed at the GC-rich Specificity Protein 1 (Sp1) promoter, correlating with the rapid downregulation of Sp protein and inhibiting proliferation and induction of apoptosis [38] (Table 1).

#### 2.1.5. Campthotecin

**Figure 2:**
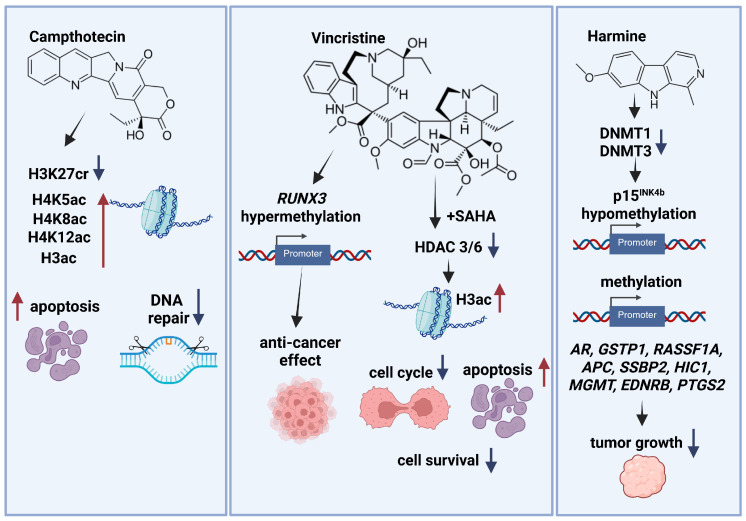
***Epigenetic effects of alkaloids on DNA methylation, histone modification, and associated regulatory enzymes—PART 2.*** Schematic representation of the epigenetic effects of selected alkaloids on cancer-related pathways. Camptothecin increases histone acetylation (H4K5ac, H4K8ac, H4K12ac, H3ac) and decreases H3K27cr, promoting apoptosis and reducing DNA repair. Vincristine, alone or in combination with the HDAC inhibitor SAHA, decreases HDAC3/6 expression and increases H3ac levels, resulting in cell cycle arrest, apoptosis, and reduced cell survival. Harmine downregulates DNMT1 and DNMT3, leading to p15^INK4b^ hypomethylation and demethylation of tumor suppressor genes (*AR*, *GSTP1*, *RASSF1A*, *APC*, *SSBP2*, *HIC1*, *MGMT*, *EDNRB*, *PTGS2*), thereby inhibiting tumor growth. **Legend: (↑)** red arrow indicates upregulation, and **(↓)** blue arrow indicates downregulation.

Camptothecin (CPT) is a pentacyclic alkaloid naturally occurring in the wood and bark of the *Camptotheca acuminata* tree, native to China [39]. This compound is a potent anticancer drug, but its low solubility and instability limit its therapeutic potential. To overcome these challenges, researchers have explored encapsulation strategies, such as using cyclodextrin-EDTA-Fe_3_O_4_ nanoparticles (CEF) [40]. Studies have shown that CPT-CEF can effectively induce apoptosis and inhibit cell growth in human colon cancer HT-29 cells by causing cell cycle arrest and activating mitochondrial apoptotic pathways. While excessive DNA damage typically leads to cell death in normal cells, cancer cells often have defective DNA repair mechanisms, making them vulnerable to therapeutic interventions [41] (Figure 2).

To understand how CPT-CEF exerts its anticancer effects, researchers analyzed gene expression in treated and untreated HT29 cells. Analyzing gene expression profiles and utilizing databases like Gene Ontology (GO) and Kyoto Encyclopedia of Genes and Genomes (KEGG), they identified three key genes involved in epigenetic control of DNA repair: high mobility group box 1 (*HMGB1*), apurinic/apyrimidinic endonuclease 1 (*APEX1*), and Polymerase (DNA-directed), epsilon 3 (*POLE3*). These genes play crucial roles in maintaining genomic (HMGB1) stability and DNA repair (APEX1, POLE3). Disruption of their function can lead to increased DNA damage and genomic instability, promoting tumorigenesis. By targeting these genes, CPT-CEF may induce apoptosis in cancer cells [42] (Figure 2).

CPT may affect histone modifications and transcriptional changes. Liao et al. [43] showed that CPT can significantly reduce the levels of H3K27cr, a histone modification associated with DNA repair in cancer cells. This effect was observed in multiple colon cancer cell lines (HCT-116, DLD-1, LoVo, and SW480) in a dose-dependent manner [43] (Figure 2).

Recent studies indicate that CPT inhibits topoisomerase 1 (Top1) by interfering with specific transcriptional regulatory pathways involving cyclin-dependent kinase (CDK) activity and polymerase II (Pol II) arrest, thereby inducing the transcription of antisense RNAs at the HIF-1α locus. HIF-1α is a transcription factor that plays a key role in the response to hypoxia, a condition in which cells are deprived of oxygen. Under hypoxic conditions, HIF-1α is stabilized and activated, leading to the expression of genes that promote cell survival and angiogenesis. CPT can also increase histone acetylation at H4K5ac, H4K8ac, H4K12ac, and histone density in transcribed *HIF-1α* regions. These changes were observed at the promoter and internal gene regions, but unmodified upstream to the HIF-1α mRNA start site. Furthermore, in satellite DNA, histone H3 acetylation (H3ac) levels were also upregulated, but limited to promoter regions [44] (Figure 2).

Microarray analysis of hepatocellular carcinoma Huh7 cells exposed to various CPT concentrations revealed the modulation of 39 miRNAs compared to untreated cells. Among the miRNAs modulated by CPT, some are known to target oncogenic activity. Notably, miR-16 and let-7, which function as tumor suppressors, are upregulated following CPT treatment in Huh7 cells. miR-16 is known to attenuate the antiapoptotic protein B-cell lymphoma 2 (Bcl-2), whereas let-7 suppresses Rat sarcoma proteins (Ras) activity in cancer cells [45,46]. CPT treatment led to the downregulation of oncogenic miRNAs (oncomirs) miR-34b and miR-222, both of which play a crucial role in anti-apoptotic mechanisms. Collectively, these findings reveal that CPT modulates biological activities by upregulating and downregulating the expression of miRNAs. CPT inhibits cell viability, upregulates miR-16 expression, leading to reduced cell migration, invasion, and clonogenic potential. This effect is mediated by the downregulation of cyclin D1, metalloproteinase 2/9 (MMP-2, MMP-9), tissue inhibitor of metalloproteinases 1 (TIMP1), and E-cadherin, along with the upregulation of cell cycle-regulated proteins p21, p27 in Huh7 cells [46].

Camptothecin upregulates the expression of miR-125 in cancer cells. It is known that miR-125b directly binds to the 3′-UTR regions of key regulatory genes, including Bcl-2 antagonist killer (*Bak1*), (Myeloid cell Leukemia-1 (*Mcl-1*), and *p53*. In CPT-treated chronic myelogenous leukemia K562 cells and cervical cancer HeLa cells, miR-125b expression was decreased, and its overexpression significantly suppressed CPT-induced apoptosis. However, miR-125b is involved in CPT-induced apoptosis by directly targeting these genes in breast cancer cells [47] (Table 1).

#### 2.1.6. Vincristine

Vincristine, a vinca alkaloid derived from the Madagascar periwinkle (*Catharanthus roseus*), is a potent anticancer drug used to treat various malignancies, including leukemias, lymphomas, and solid tumors. It has been shown to exhibit a wide range of biological activities, including cell cycle arrest and apoptosis induction, making it an effective chemotherapeutic agent [48].

Recent studies have highlighted vincristine’s ability to modulate epigenetic processes. Moon et al. [49] demonstrated that vincristine treatment led to demethylation of the runt-related transcription factor 3 (*RUNX3*) gene in DLD-1 colorectal adenocarcinoma cells, restoring RUNX3 expression. Since RUNX3 is a tumor suppressor gene frequently silenced by DNA methylation in colorectal cancer, its reactivation suggests a potential mechanism for vincristine’s anticancer effects. Quantitative methylation-specific polymerase chain reaction (QMSP) and real-time PCR analyses confirmed hypermethylation of *RUNX3* in a significant proportion of colorectal cancer samples, along with decreased RUNX3 mRNA expression. These findings suggest that vincristine may exert its anticancer effects, in part, by reversing epigenetic silencing of tumor suppressor genes [49] (Figure 2).

Chao et al. [50] showed that combined vincristine with suberoylanilide hydroxamic acid, a histone deacetylase (HDAC) inhibitor, effectively inhibited HDAC3 and HDAC6 expression. This combination also enhanced histone acetylation and induced a time-dependent reduction in HDAC activity. Functionally, this dual treatment significantly reduced T-lymphoblastoid MOLT-4 cell survival by inducing M-phase cell cycle arrest and activating both intrinsic and extrinsic apoptotic pathways, demonstrating a synergistic interaction between vincristine and the HDAC inhibitors [50] (Figure 2).

Additionally, studies have indicated that vincristine can modulate miRNA levels. In primary leukemia cell lines (JM1, Sup-B15, and NALM-6), vincristine treatment resulted in the suppression of miR-181a expression, whereas no such effect was observed in relapsed leukemia cell lines (NALM-16 and REH). Vincristine exposure resulted in the suppression of both cellular and exosomal miR-181a expression, leading to induced apoptosis in primary leukemia cells. Cell-derived components, such as exosomes and extracellular matrix, play a crucial role in shaping the tumor microenvironment in hematological malignancies. These findings suggest that vincristine’s impact on miRNA expression may contribute to its therapeutic efficacy [51] (Table 1).

#### 2.1.7. Harmine

Harmine, a naturally occurring β-carboline alkaloid found in the seeds of the plant *Peganum harmala*, has shown promising anticancer properties in various studies. This compound has attracted significant attention due to its potential to target multiple cancer hallmarks. Harmine can arrest the cell cycle at various phases, trigger programmed cell death, and suppress angiogenesis in cancer cells [52].

Recent studies have hinted at the potential of harmine to alter epigenetic mechanisms in cancer cells. In NB4 leukemic cells, harmine led to a decrease in DNMT1 mRNA expression, which was accompanied by the reactivation of the p15^INK4B^ tumor suppressor gene and hypomethylation of its promoter, which ultimately contributed to tumor growth inhibition [53].

Studies have indicated that harmine directly binds to DNA methyltransferase. Harmine’s binding to the adenine cavity of the SAM-binding pocket in DNMT3B suggests its potential to interact with other proteins, such as protein kinases and methyltransferases, which rely on ATP and SAM cofactors. Harmine may reactivate hypermethylated genes by inhibiting DNMTs and reducing promoter methylation of androgen receptor (*AR*), glutathione S-transferase Pi 1 (*GSTP1*), Ras association domain family member 1A (*RASSF1A*), Adenomatous Polyposis Coli (*APC*), single-strand binding protein 2 (*SSBP2*), hypermethylated in cancer 1 (*HIC1*), O-6-methylguanine-DNA methyltransferase (*MGMT*), endothelin receptor type B (*EDNRB*), prostaglandin-endoperoxide synthase 2 (*PTGS2*) and suppress prostate cancer cell growth [54] (Figure 2).

### 2.2. Terpenoids

Terpenoids, also known as isoprenoids, constitute the largest and most diverse class of natural products, widely found in plants, animals, and microorganisms. These compounds are derived from the five-carbon building block isoprene. They are classified based on the number of isoprene units they contain (hemiterpenoids, monoterpenoids, sesquiterpenoids, diterpenoids, sesterterpenoids, triterpenoids, tetraterpenoids, polyterpenoids). Terpenoids exhibit a remarkable structural diversity, ranging from simple linear molecules to complex cyclic structures [55].

In recent years, terpenoids have also gained attention for their potential health benefits, particularly for their anticancer properties. Some terpenoids have been shown to inhibit the growth and spread of cancer cells in laboratory and animal studies [56]. The mechanisms by which terpenoids exert their anticancer effects are complex and varied, but they involve inducing apoptosis [57], inhibiting angiogenesis [56], suppressing the growth of cancer stem cells [56], and modulating the immune response against cancer [56].

#### 2.2.1. Hinokitiol

**Figure 3:**
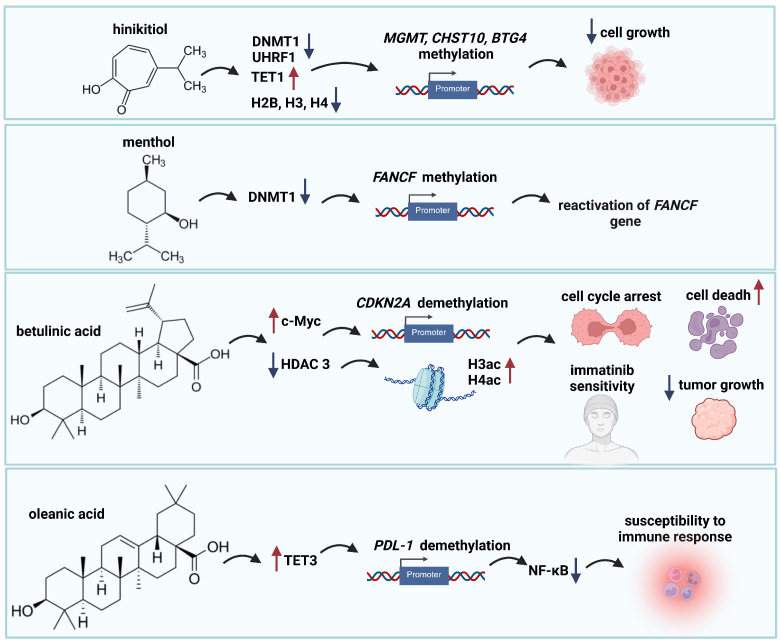
**Epigenetic mechanisms underlying the anticancer effects of terpenoid.** Hinokitiol, menthol, betulinic acid, and oleanolic acid modulate key epigenetic regulators involved in DNA methylation and histone modification. Hinokitiol downregulates DNMT1 and UHRF1 while upregulating TET1, leading to altered methylation of *MGMT*, *CHST10*, and *BTG4* promoters and inhibition of cell growth. Menthol suppresses DNMT1 activity, resulting in *FANCF* promoter demethylation and gene reactivation. Betulinic acid decreases HDAC3 expression and promotes *CDKN2A* demethylation and histone acetylation (H3ac, H4ac), which enhance imatinib sensitivity, induce cell cycle arrest, and promote apoptosis. Oleanolic acid upregulates TET3 and facilitates *PD-L1* promoter demethylation, reducing NF-κB signaling and increasing susceptibility to immune response. **Legend: (↑)** red arrow indicates upregulation, and **(↓)** blue arrow indicates downregulation.

Hinokitiol belongs to monoterpenoids, extracted from the heartwood of the hinoki cypress tree. This compound has attracted significant attention for its potential anticancer properties through the regulation of epigenetic changes [58]. Treatment of colon cancer HCT-116 cells with hinokitiol resulted in a time- and dose-dependent decrease in *DNMT1* mRNA and protein expression. This compound also reduced the expression of ubiquitin-like with PHD and RING finger domains 1 (UHRF1), a protein that interacts with DNMT1 to maintain DNA methylation. Concurrently, hinokitiol increased the levels of Tet methylcytosine dioxygenase 1 (TET1) protein and 5-hydroxymethylcytosine (5hmC), indicating an increase in DNA demethylation. These findings suggest that hinokitiol may promote DNA demethylation in colon cancer cells by downregulating DNMT1 and upregulating TET1 activity. The demethylation effect was further supported by the reactivation of several genes silenced by DNA methylation in colon cancer cells, such as *MGMT*, carbohydrate sulfotransferase 10 (*CHST10*), and B-cell translocation gene 4 (*BTG4*), leading to inhibited cancer cell growth [59] (Figure 3).

Microarray analysis revealed that hinokitiol significantly downregulated the expression of multiple replication-dependent histone genes in oral squamous cell carcinoma (OSCC) cells. This downregulation was confirmed for specific histone genes (*HISTH2BM* and *HISTH3J*) and a decrease in the protein levels of H2B, H3, and H4 histones in OSCC cells treated with hinokitiol. The reduction in histone protein levels led to increased sensitivity of chromatin to micrococcal nuclease digestion, suggesting alterations in chromatin structure. Hinokitiol also suppressed the expression of Fas-activated serine/threonine kinase (FLASH) and nuclear protein, coactivator of histone transcription (NPAT), key transcriptional activators of histone genes. Chromatin immunoprecipitation (ChIP) assays revealed changes in the binding of NPAT and FLASH to histone gene promoters in response to hinokitiol treatment. Hinokitiol significantly suppressed the activity of histone gene promoters, particularly the H2B/r, H3/a, and H4/e promoters. Hinokitiol demonstrates efficacy against oral squamous cell carcinoma cell growth by suppressing the expression of multiple types of histones (pan-histones) [60] (Figure 3).

Studies also showed that hinokitiol treatment led to an increase in the expression of miR-494-3p, a microRNA known to target and downregulate BMI1 expression. Inhibition of miR-494-3p partially reversed the inhibitory effects of hinokitiol on B lymphoma Mo-MLV insertion region 1 homolog (*BMI1*) expression and mammosphere formation in breast cancer cells. Luciferase reporter assays demonstrated that miR-494-3p directly targets the 3′-untranslated region (3′-UTR) of the *BMI1* mRNA, leading to its degradation or translational inhibition. These findings suggest that hinokitiol exerts its anticancer effects in breast cancer by modulating the miRNA-mediated regulation of *BMI1*, a critical factor in maintaining the stem-like properties of cancer cells [61] (Table 2).

#### 2.2.2. Menthol

Menthol is an organic compound, specifically an alcohol belonging to the cyclic monoterpene. It is primarily found in peppermint oil and gives mint its characteristic cooling sensation and strong aroma [62].

Menthol can inhibit the growth of various human cancer cell lines, including neuroendocrine tumor cells, melanoma cells, promyelocytic leukemia cells, colon cancer cells, liver tumor cells, and glioblastoma cells [63]. Menthol decreases DNA methylation levels at specific CpG sites within the promoter region of the Fanconi Anemia Complementation Group F (*FANCF*) gene in cervical cancer cells (SiHa line). Researchers observed hypomethylation at all 15 CpG sites, resulting in a complete loss of FANCF methylation. This effect was likely mediated by the inhibition of DNMT1 activity, an enzyme responsible for maintaining DNA methylation patterns [63] (Figure 3).

#### 2.2.3. Betulinic Acid

Betulinic acid (BA), a pentacyclic triterpene, is found in various plants, including birch, eucalyptus, and plane trees. It exhibits anti-inflammatory, antibacterial, antiviral, antidiabetic, antimalarial, anti-HIV, and antitumor effects [64]. Studies indicated that betulinic acid may regulate epigenetic modifications in cancer cells. BA selectively modulates DNA methylation at key gene promoters in the Kaiso signaling pathway: the oncogene *c-Myc* and the tumor suppressor gene cyclin-dependent kinase inhibitor 2A (*CDKN2A*). It silences c-Myc while enhancing the demethylation and increased expression of *CDKN2A*. These effects, observed in breast cancer cell lines MCF-7 and MDA-MB-231, lead to cell cycle arrest and cancer cell death [65] (Figure 3).

Studies demonstrate that betulinic acid can effectively restore the sensitivity of imatinib-resistant leukemia cells (K562R) by targeting HDAC3. Specifically, betulinic acid promotes the degradation of HDAC3, leading to an increase in the histone acetylation (H3ac and H4ac), altering gene expression patterns and reversing imatinib resistance. Furthermore, the study observed a significant reduction in tumor growth in mice when imatinib and betulinic acid were administered in combination [66] (Figure 3).

In breast and colon cancer cells, BA reduces the levels of miRNA-27a. This miRNA directly targets *ZBTB10*, a transcriptional repressor. By decreasing miRNA-27a, betulinic acid increases ZBTB10 levels, leading to the suppression of Sp1, Sp3, and Sp4, which are transcription factors involved in cell growth and proliferation. This ultimately leads to a significant reduction in tumor volume and weight in animal models [67,68]. Furthermore, in breast cancer cells, betulinic acid downregulates miR-20a, miR-106a, and miR-106b. These miRNAs also target Sp1, Sp3, and Sp4, as well as enhancer of zeste homolog 2 (EZH2), an enzyme involved in gene silencing. By suppressing these miRNAs, BA indirectly increases transcription factor ZBTB4, further inhibiting tumor growth [69]. In hepatocellular carcinoma (HCC), betulinic acid reduces the levels of metastasis-associated lung adenocarcinoma transcript 1 (MALAT1), a long non-coding RNA that acts as a “sponge” for miRNA-22-3p. By reducing MALAT1, betulinic acid enhances miRNA-22-3p activity, leading to downregulation of X-linked inhibitor of apoptosis protein (XIAP) and survivin, two proteins that support cancer cell survival. As a consequence, there is an increase in apoptosis of hepatocellular cancer HCC cells [70] (Table 2).

Treatment with BA significantly reduced tumor size in a dose-dependent manner, accompanied by decreased cell proliferation and increased apoptosis of pancreatic cancer cells. This effect was linked to the downregulation of miR-365 expression, which led to increased *BTG2* expression and the inhibition of the IL-6/AKT/STAT3 signaling pathway. These findings suggest that BA exerts its anticancer effects in pancreatic cancer by modulating the miR-365/BTG2 axis, ultimately suppressing tumor growth and progression [71] (Table 2).

#### 2.2.4. Oleanolic Acid

Oleanolic acid (OA) is a naturally occurring pentacyclic triterpenoid found in a wide variety of plants, including olive leaves, rosemary, and some fruits. It has been studied for its potential therapeutic properties in various conditions [72]. Oleanolic acid is being investigated as a potential therapeutic agent for various diseases, including cancer, diabetes, and inflammatory conditions [73]. Oleanolic acid has shown promise as a potential therapeutic agent in gastric cancer by modulating the immune response. In this context, OA inhibits the expression of PD-L1, a protein that helps cancer cells evade immune response. This effect is mediated through the suppression of DNA demethylation. Specifically, OA disrupts the IL-1β/NF-κB/TET3 pathway. TET3 then promotes DNA demethylation in the PD-L1 gene promoter, ultimately increasing PD-L1 expression. However, OA inhibits the activation of the NF-κB pathway, preventing the induction of TET3 expression and subsequent DNA demethylation. This results in decreased PD-L1 expression, making the cancer cells more susceptible to immune response [74] (Figure 3).

OA activates miR-122 expression, leading to the inhibition of hepatocellular carcinoma HCC cell migration and invasion. Mechanistically, OA exerts its anti-metastatic effects by miR-122 modulating the expression of EMT markers, including E-cadherin, β-catenin, N-cadherin, and vimentin [75] (Table 2).

Studies have reported that miR-98-5p expression was significantly downregulated in gastric cancer (GC) tissues. miR-98-5p overexpression inhibited Treg (regulatory T-cells) and Th17 (T helper 17 cells) cell differentiation in vitro. Furthermore, oleanolic acid was shown to regulate Treg/Th17 balance by upregulating miR-98-5p expression. OA-mediated upregulation of miR-98-5p led to downregulation of IL-6, a key cytokine involved in Th17 cell differentiation. These findings suggest that OA may exert anti-tumor effects in GC by modulating the Treg/Th17 balance through the miR-98-5p/IL-6 axis [76] (Table 2).

OA has been demonstrated to effectively inhibit the proliferation of chronic myeloid leukemia (CML) cells and induce apoptosis. Studies showed that OA treatment significantly downregulates miR-18a-5p. However, miR-18a-5p directly targets the 3′-UTR of serine/threonine kinase 4 (STK4) mRNA, which suggests that OA may exert its effects by suppressing miR-18a-5p, thereby releasing the inhibition of STK4 expression. miR-18a-5p overexpression partially reversed the anti-proliferative and pro-apoptotic effects of OA and counteracted the effects of STK4 overexpression [77] (Table 2).

#### 2.2.5. Ursolic Acid

**Figure 4:**
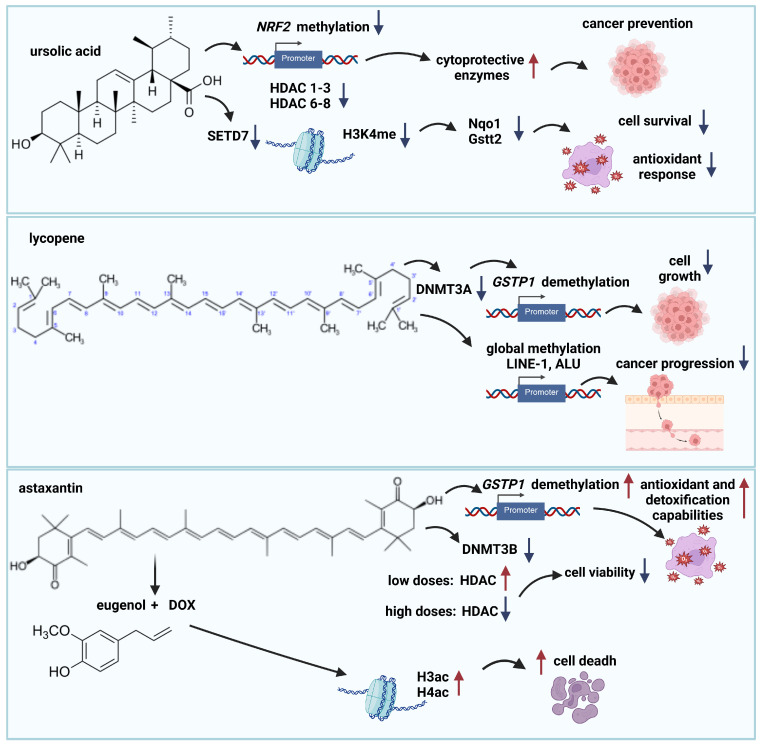
**Epigenetic regulatory effects of natural compounds in cancer prevention and progression.** Ursolic acid, lycopene, and astaxanthin regulate key epigenetic enzymes involved in DNA methylation and histone modification, thereby influencing cancer-related pathways. Ursolic acid decreases the expression of HDAC1–3 and HDAC6–8 and reduces SETD7 levels, leading to lower H3K4 methylation and *NRF2* promoter demethylation. These changes enhance cytoprotective enzyme expression (*Nqo1*, *Gstt2*), suppress oxidative stress, and contribute to cancer prevention. Lycopene downregulates DNMT3A, resulting in *GSTP1* promoter demethylation and decreased global methylation of repetitive elements (*LINE-1*, *ALU*), which collectively inhibit cancer progression. Astaxanthin, alone or in combination with eugenol and doxorubicin (DOX), reduces DNMT3B expression and promotes *GSTP1* demethylation. It also increases histone acetylation (H3ac, H4ac) and exhibits dose-dependent regulation of HDACs, leading to enhanced antioxidant defense at low doses and induction of cell death at higher concentrations. **Legend: (↑)** red arrow indicates upregulation, and **(↓)** blue arrow indicates downregulation.

Ursolic acid (UA), a pentacyclic triterpenoid found in a wide variety of plants, including apple peel, rosemary, and thyme, exhibits a wide range of pharmacological activities, including anti-inflammatory, antioxidant, anti-apoptotic, and anticancer properties [78]. Research suggests it may have chemopreventive effects against skin cancer, particularly by influencing epigenetic mechanisms. In JB6 P+ skin cancer cells, UA decreases DNA methylation within the *Nrf2* promoter region. UA significantly downregulated the expression of several HDACs, specifically those belonging to Class I (HDAC1, 2, 3, and 8) and Class II (HDAC6 and 7). This decrease in HDAC expression led to a corresponding reduction in HDAC activity. With decreased HDAC activity, there was a significant increase in the H3ac. This epigenetic modulation is achieved through reduced expression of DNA methyltransferases and histone deacetylases, coupled with increased histone acetylation. Consequently, UA enhances the expression of cytoprotective enzymes, ultimately suppressing tumor promoter-induced cell transformation. These findings highlight the potential of UA as an epigenetic regulator in skin cancer prevention [79]. Further studies have explored the role of UA in regulating the antioxidant response in prostate cells. Wang et al. [80] found that UA significantly increased the expression of SET domain containing 7 (Setd7) in prostate cancer cells. Setd7 plays a crucial role in activating the Nrf2-ARE signaling pathway, a key pathway involved in cellular antioxidant defense. Inhibiting Setd7 significantly reduced the expression of Nrf2-regulated antioxidant genes such as NAD(P)H quinone dehydrogenase 1 (*Nqo1*) and glutathione S-transferase theta 2 (*Gstt2*) through enrichment of H3K4me1 in the promoter regions of these genes. These results suggest that Setd7 is a key Nrf2 regulator, with significant effects on antioxidant response, DNA protection, and cell survival [80] (Figure 4).

In breast cancer research, UA has shown the ability to reverse paclitaxel (PTX) resistance. UA upregulates miR-149-5p expression, which leads to the downregulation of myeloid differentiation primary response gene 88 (MyD88), ultimately inhibiting the PI3K/AKT pathway and enhancing PTX-induced cell death [81]. However, UA also exerts anticancer effects on breast cancer stem cells (bCSCs). It downregulates the miR-499a-5p expression. This miRNA is significantly upregulated in bCSCs compared to parental breast cancer cells, suggesting its potential role in maintaining stem-like properties. Overexpression of miR-499a-5p in breast cancer cells promoted cell proliferation, upregulated the expression of oncogenes such as *FOS*, cyclin D3 (*CCND3*), and secreted phosphoprotein 1 (*SPP1*), and simultaneously downregulated tumor suppressor genes, including mouse double minute 2 (*MDM2*), von Hippel–Lindau (*VHL*), and polymerase beta (*POLB*). UA-mediated downregulation of miR-499a-5p contributes to its anti-proliferative effects. Furthermore, UA inhibits T-cell factor/lymphoid enhancer factor (TCF/LEF) activity, suggesting its ability to suppress the Wnt/β-catenin pathway. Since miR-499a-5p is linked to Wnt pathway activation, this suggests a dual mechanism of action. Moreover, UA restores the expression of Wnt antagonists (secreted frizzled-related protein 4, sFRP4; Dickkopf-related protein 1, DKK1), further contributing to the inhibition of the Wnt/β-catenin pathway and decrease the expression of key CSC markers (cluster of differentiation- CD44, aldehyde dehydrogenase 1, ALDH1; ATP-Binding cassette subfamily C member 2, ABCC2; and ATP-Binding cassette subfamily G member 2, ABCG2) [82] (Table 2).

Zhang et al. [83] reported that UA significantly increased the levels of miR-200a and miR-200c in two colorectal cancer cell lines (HCT-116 and HCT-8). UA may effectively inhibit the growth and spread of colorectal cancer cells by regulating the TGF-β1/ZEB1/miR-200c feedback loop. By inhibiting transforming growth factor beta 1 (TGF-β1) and increasing miR-200 expression, UA helps restore the balance that prevents aggressive cancer cell behavior [83] (Table 2).

#### 2.2.6. Lycopene

Lycopene, a carotenoid pigment found in tomatoes and other red fruits and vegetables, has been shown to have potential anticancer properties. One mechanism by which lycopene may exert its anticancer effects is through epigenetic modifications, particularly DNA methylation.

In androgen-independent PC-3 prostate cancer cells, lycopene treatment was shown to significantly downregulate the expression of DNA methyltransferase 3A (DNMT3A). This downregulation may contribute to demethylation of the *GSTP1* promoter, reactivation of its expression, and inhibition of cancer cell growth. Additionally, in androgen-dependent LNCaP cells, lycopene treatment resulted in a significant reduction in global methylation levels in long interspersed nuclear element (LINE-1) and ALU element CpGs, compared to untreated control cells. These repetitive elements are typically heavily methylated in healthy tissues; however, their hypomethylation has been associated with cancer progression [84] (Figure 4).

Lycopene has also been linked to the regulation of specific microRNAs, such as miR-let-7f-1, which plays a role in controlling cell proliferation and apoptosis. Recent research indicates that lycopene may upregulate miR-let-7f-1 and bind to AKT2. By binding to the AKT2 mRNA, miR-let-7f-1 promotes its degradation, leading to reduced AKT2 protein levels. This decrease in AKT2 activity subsequently inhibits cell proliferation and promotes apoptosis [85] (Table 2).

#### 2.2.7. Astaxhantin

Astaxanthin (AST), a potent carotenoid known for its vibrant red color, has garnered significant attention for its potential anticancer properties. This powerful antioxidant possesses a unique molecular structure that enables it to effectively neutralize harmful free radicals, thereby reducing oxidative stress and DNA damage, both of which are implicated in cancer development [86].

Numerous studies have demonstrated the anticancer effects of astaxanthin across various cancer types, including prostate, breast, lung, colon, and skin cancers [87]. Its exceptional antioxidant capacity allows it to scavenge harmful free radicals, protecting cells from oxidative damage and lowering the risk of cancer initiation and progression [88]. In addition, astaxanthin can suppress chronic inflammation, induce cell cycle arrest, trigger programmed cell death in cancer cells, and inhibit angiogenesis [89,90,91].

Evidence suggests that AST primarily exerts its effects through epigenetic mechanisms, particularly DNA methylation. By demethylating specific CpG sites within the *GSTP1* promoter, AST can increase its expression. This, in turn, enhances the antioxidant and detoxification capabilities of cancer cells by increasing GSTP1 protein levels [92] (Figure 4).

Astaxanthin can also modulate the epigenetic landscape by influencing the expression of DNA methyltransferases and histone deacetylases. Specifically, astaxanthin reduce the expression of DNMT3B. Interestingly, low doses of astaxanthin increase HDAC activity, while higher doses inhibit it. This suggests a complex dose-dependent relationship between astaxanthin and HDAC activity [92] (Figure 4).

Studies have also shown that AST, when combined with treatments like doxorubicin and eugenol, can significantly increase H3ac and H4ac, and also upregulate the expression of histone acetyltransferases in breast cancer cells. This histone modification enhanced DOX-induced cell death [93] (Figure 4).

Additionally, AST inhibits c-Myc expression and indirectly upregulates miR-29a-3p and miR-200a. The upregulation of miR-29a-3p enables this microRNA to bind to the 3′-UTR of MMP2 mRNA, while the restoration of miR-200a levels leads to suppression of ZEB1. By inhibiting c-Myc expression at the transcriptional level, AST indirectly upregulates miR-29a-3p and miR-200a, ultimately suppressing the metastatic potential of colon cancer cells [94] (Table 2).

#### 2.2.8. Lutein

Lutein is a natural pigment found in many fruits and vegetables, responsible for the yellow, orange, and red colors in some plants. Beyond its role in giving plants their vibrant hues, lutein provides significant health benefits, particularly for eye health. As an antioxidant, it helps protect cells throughout the body from damage caused by free radicals—unstable molecules linked to aging and various diseases such as cancers. Research suggests that lutein may inhibit the growth and spread of certain types of cancer cells, including breast, cervical, and colon cancer.

Researchers found that treating breast cancer cells (MCF-7 and T47D) with lutein significantly increased levels of miR-590-3p in a dose-dependent manner. Overexpression of miR-590-3p inhibited the growth of breast cancer cells. Furthermore, combining lutein treatment with miR-590-3p mimic (which artificially increases miR-590-3p levels) significantly enhanced the anti-proliferative effects of lutein, indicating a synergistic interaction. The most compelling finding is that simultaneously inhibiting caspase 9 (CASC9) and overexpressing miR-590-3p in the presence of lutein resulted in a greater suppression of breast cancer cell growth than either intervention alone. This strongly suggests that the CASC9/miR-590-3p axis plays a critical role in mediating the anticancer effects of lutein [95] (Table 2).

### 2.3. Polyphenols

Polyphenols, characterized by the presence of multiple phenol units within their structure, exhibit potent antioxidant, anti-inflammatory, and antibacterial activities. These effects are attributed to their ability to neutralize free radicals, modulate immune responses, and interfere with microbial cell structures [96]. Given the growing consumer preference for natural alternatives to synthetic additives, along with the escalating issue of antibiotic resistance, researchers are increasingly focusing on the potential of polyphenols as natural therapeutic agents. Their antioxidant and anti-inflammatory properties offer promising avenues for the prevention and management of neurodegenerative diseases, cardiovascular diseases, and cancers [96,97]. Polyphenols represent a large and diverse group of plant-based compounds, classified into several categories: flavonoids (flavones, flavonols, flavanones, flavanols, anthocyanidins) [98], phenolics acids (hydroxycinnamic acids, hydroxybenzoic acids), lignans, stilbenes, and tanins [98,99].

#### 2.3.1. Apigenin

Apigenin is a type of flavonoid, a plant-based compound found in various fruits and vegetables, most notably parsley, celery, chamomile, onions, and apples [100]. Apigenin may exert its effects by interacting with various signaling pathways involved in cancer development, such as the PI3K/AKT/mTOR pathway, NF-κB, signal transducer and activator of transcription 3 (Stat3) pathways, angiogenesis, apoptosis, cell cycle, and inflammation. Recent research also suggests that apigenin can induce changes in the epigenetic modifications observed in cancer cells [101].

In triple-negative breast cancer, apigenin significantly inhibited the activity of HDACs (HDAC 1/3) and DNMTs enzymes and increased HAT activity in a dose-dependent manner. Mechanistically, apigenin directly inhibited HDAC activity by binding to the enzyme’s active site, as confirmed by molecular docking and dynamics simulations. This led to changes in the expression of genes involved in apoptosis, including increased expression of pro-apoptotic proteins (Bax and Bid) and decreased expression of anti-apoptotic proteins (Bcl-2). Additionally, apigenin modulated microRNA expression by reducing the oncogenic miR-21 and increasing the tumor-suppressor miR-200b [102].

In prostate cancer cells, apigenin also significantly reduced the expression and activity of HDAC1 and HDAC3, leading to increased H3ac, H4ac. Specifically, it elevated H3ac levels at the p21/Waf1 promoter region in PC-3 cells. Furthermore, apigenin significantly inhibited HDAC activity and promoted apoptosis in the xenografts. It also reduced HDAC1 and HDAC3 protein expression in a dose-dependent manner, while increasing p21/Waf1 and Bax protein levels, decreasing Bcl2, and shifting the Bax/Bcl2 ratio in favor of apoptosis [103] (Table 3).

Apigenin can interfere with various signaling pathways involved in cancer cell growth, survival, and metastasis. In HCT116 colon cancer cells, apigenin treatment significantly increased the expression of miR-215-5p, which directly targets and inhibits the expression of E2F1 and E2F3, which are key transcription factors involved in cell cycle progression. Downregulation of these genes by miR-215-5p contributes to apigenin-induced cell cycle arrest at the G0/G1 phase [104].

In glioma cancer cells, apigenin increases miR-16 expression, which leads to the downregulation of Bcl-2, NF-κB, and MMP-9. This cascade of events promotes apoptosis by reducing Bcl-2 and lessens the invasive potential of cancer cells by inhibiting NF-κB and MMP-9, suggesting a promising therapeutic role for apigenin [105].

Apigenin has also shown potential as a chemosensitizer, particularly in overcoming drug resistance in cancer cells. In doxorubicin-resistant BEL-7402 liver cancer cells, this sensitization appears to be mediated through a miR-101-dependent mechanism. These resistant cells exhibit a characteristic downregulation of miR-101 and upregulation of NRF2, a protein that confers resistance to doxorubicin. Apigenin treatment restores miR-101 expression, enabling it to bind to the 3′-UTR of NRF2 mRNA, triggering post-transcriptional gene silencing. The downregulation of NRF2 by miR-101 is essential for apigenin’s chemosensitizing effect, as it effectively reverses the resistance mechanism. By reducing NRF2 levels, apigenin allows doxorubicin to regain its cytotoxic efficacy, ultimately leading to increased cancer cell death [106] (Table 4).

Given the extensive body of research exploring the effects of polyphenols on epigenetic modifications, a concise summary of their reported epigenetic actions and the subsequent cellular outcomes in cancer cells is compiled in the table below (Table 3 and Table 4).

Furthermore, apigenin significantly increases the expression of miR-103a-3p in glioma cells. This upregulation of miR-103a-3p leads to the downregulation of neural precursor cell-expressed developmentally down-regulated protein 9 (NEDD9), which subsequently inhibits the FAK/AKT signaling pathway, ultimately resulting in reduced cell proliferation, migration, invasion, and epithelial–mesenchymal transition (EMT). This represents a novel mechanism by which apigenin may exert its anticancer effects in glioma [107] (Table 4).

#### 2.3.2. Luteolin

Luteolin (LUT) is a natural flavonoid found in many plants, especially common vegetables, fruits, and herbs. Rich dietary sources of luteolin include celery, parsley, broccoli, onion leaves, carrots, peppers, cabbage, apple skins, and chrysanthemum flowers. Luteolin helps protect plants from stress caused by microbes, insects, and UV radiation. Beyond its role in plants, luteolin has demonstrated potential health benefits for humans and animals, primarily due to its antioxidant, estrogenic regulatory, and antimicrobial properties. Like other flavonoids, luteolin has been identified as a potentially cancer-preventive agent [108].

Studies show that LUT influences the Nrf2 pathway through epigenetic modifications in colorectal cancer cells (HCT-116). LUT decreases the CpG methylation of the Nrf2 promoter and increases Nrf2 gene expression. This demethylation is associated with the ability of LUT to suppress the protein expression and enzyme activity of DNMT1, DNMT3A, and DNMT3B in HCT116 cells. Following a 5-day treatment with LUT, protein levels of HDAC1-3, 6- 7 were all significantly reduced. Notably, the reduction in HDAC3, HDAC6, and HDAC7 expression exhibited a clear concentration-dependent relationship. Therefore, the regulation of DNMTs and HDACs by LUT results in the inhibition of cell growth and colony formation [109]. In colon cancer cells, luteolin increases TET1 expression, leading to demethylation of the Nrf2 promoter and upregulation of Nrf2 expression. Elevated Nrf2 levels enhance its interaction with p53, resulting in increased expression of antioxidant enzymes (e.g., heme oxygenase-1) and changes in apoptosis-related proteins (Bax, Bcl-2, caspase 9). This combined effect contributes to the induction of apoptosis in colon cancer cells [110].

Luteolin treatment also effectively increases both opioid-binding protein/cell adhesion molecule (OPCML) mRNA and protein levels in a dose-dependent manner. The OPCML gene, a member of the IgLON family, functions as a tumor suppressor and is often silenced in various cancers, including breast cancer, due to hypermethylation of its promoter region. Luteolin downregulates DNMT1 expression and activity, reduces global methylation levels, and reverses *OPCML* gene promoter methylation. Furthermore, the transcription factor Sp1, known to regulate cell cycle-related molecules, is also implicated in DNMT1 regulation. Sp1 binds to the *DNMT1* promoter and enhances its expression. Research shows that luteolin treatment significantly reduces Sp1. Importantly, Sp1 overexpression partially reverses the inhibitory effect of luteolin on DNMT1 expression and counteracts the luteolin-induced upregulation of OPCML. Therefore, luteolin effectively inhibits tumor cell growth and induces apoptosis [111].

Luteolin has also been identified as a specific inhibitor of EZH2, preferentially targeting its catalytic site. Treatment of human prostate cancer cell lines DU145 and 22Rv1, known for high constitutive EZH2 expression, with luteolin significantly reduced EZH2 and suppressor of zeste 12 (SUZ12) protein levels in a dose- and time-dependent manner. However, luteolin did not affect the protein expression of embryonic ectoderm development (EED) and retinoblastoma-associated proteins 46/48 (RbAp46/48), other members of the Polycomb Repressive Complex 2 (PRC2). Furthermore, luteolin reduced H3K27me3 levels and enzymatic activity of PRC2 complex in a dose- and time-dependent manner, without affecting total H3 protein levels. These effects increased in the expression of downstream tumor suppressor genes, including E-cadherin (*CDH1*), *slit guidance ligand 2* (*SLIT2*), and *TIMP3*, and thereby reducing prostate cancer cell proliferation and invasion [112].

Luteolin is also a potent and specific inhibitor of p300 acetyltransferase activity. Molecular docking studies reveal that luteolin binds to the acetyl-CoA binding site of p300, interacting with residues (W1436 and C1438) crucial for its activity. The presence of an additional hydroxyl group (-OH) in luteolin allows for these critical interactions, explaining its greater potency. In oral cancer cells, luteolin treatment results in reduced H3K9ac and H3K14ac, while histone H4 acetylation is less affected. Immunohistochemical analysis of tumor samples confirms decreased H3K9ac and H3K14ac and reduced Ki67 levels (a proliferation marker) in the luteolin-treated group. Chromatin immunoprecipitation assays show that luteolin reduces H3K9 acetylation on the promoters of cancer-promoting genes (*IL6*; Adenosine A1 Receptor, *ADORA1*; teneurin transmembrane protein 1, TENM1), directly linking its lysine acetyltransferase KAT inhibitory activity to gene expression changes and inhibiting head and neck squamous cell carcinoma progression [113].

In HL-60 cells, luteolin treatment increases histone H3 acetylation over time and induces apoptosis. This occurs through c-Jun activation, which subsequently upregulates Fas and FasL expression, leading to caspase-8-mediated apoptosis [114].

Wu et al. [115] found that luteolin inhibits the proliferation and metastasis of androgen receptor-positive triple-negative breast cancer cells. This inhibition occurs through epigenetic regulation of MMP9 expression, specifically by reducing AKT/mTOR-inducing H3K56ac and H3K27ac levels [115] (Table 3).

Luteolin treatment effectively lowers miR-301-3p levels in pancreatic cancer cells. Since miR-301-3p directly targets and suppresses caspase-8, and its downregulation is linked to TNF-related apoptosis-inducing ligand (TRAIL) resistance in pancreatic cancer PANC-1 cells. Consequently, by downregulating miR-301-3p, luteolin restores caspase-8 levels, sensitizing cancer cells to apoptosis, notably TRAIL-induced apoptosis [116].

Luteolin plays a role in regulating gene expression related to lung cancer metastasis through its influence on miR-106a-5p. Luteolin treatment leads to an increase in the levels of miR-106a-5p, which acts as a regulator of twist family BHLH transcription factor 1 (*TWIST1*) and *MMP2* genes. *TWIST1* and *MMP2* are known to be involved in processes gastric cancer’s development [117].

Some research indicates that luteolin can increase miR-34 expression, which in turn can suppress tumor growth by targeting genes involved in cell proliferation and survival. Cells treated with both luteolin and an anti-miR-34a oligonucleotide (AMO, which inhibits miR-34a), the levels of p53 and p21 were decreased, while the phosphorylation levels of MEK and ERK were increased. However, decreasing HK1 expression in luteolin-resistant cells partially restored their sensitivity to luteolin, mirroring the effect of increasing miR-34a levels. This strongly suggests that miR-34a mediates luteolin sensitivity by targeting HK1 through directly binding to the 3′-UTR of HK1. These observations suggest that miR-34a modulates luteolin sensitivity, at least in part, by influencing these critical signaling pathways involved in cell cycle, apoptosis, and cell proliferation. Mice bearing tumors treated with luteolin and a miR-34a agomir exhibited smaller tumor sizes and weights compared to mice treated with luteolin and a miR-34a antagomir. This in vivo evidence reinforces the notion that miR-34a enhances the effectiveness of luteolin in suppressing tumor growth [118]. Researchers found that mice receiving both luteolin and a miR-34a agomir had smaller tumors than mice receiving luteolin along with a miR-34a antagomir. This strongly suggests that increasing miR-34a makes luteolin more effective at shrinking tumors [118] (Table 4).

#### 2.3.3. Chrysin

Chrysin, a flavone found in honey, bee propolis, and passion flower, has shown promise as a treatment for cancer. It is known for its antioxidant, antiviral, and anticancer properties, and can disrupt cancer cell growth through blocking cell division, inhibiting angiogenesis, and triggering cell death. However, chryspin impacts the epigenome of various cancer cells. Chrysin reduces the activity of DNA methyltransferases, histone deacetylases, and histone acetyltransferases as well as decreases global DNA methylation in HeLa cells. Chrysin alters the expression levels of several genes involved in chromatin modification. It downregulates several groups of epigenetic regulators, including DNA methyltransferases (DNMT1, DNMT3A, and DNMT3B), histone-modifying enzymes such as histone deacetylases (HDACs), histone acetyltransferase (HAT1), and protein arginine methyltransferase (PRMT8), histone methyltransferase SEDT2, as well as cell-cycle–related kinases (AURKA and AURKB) and other chromatin-associated factors like *WHSC1*, *EHM2*, *ESCO2*, and *CIITA*. Importantly, it also decreases methylation at the promoter regions of several key tumor suppressor genes, such as *APC*, *BRCA1*, *CDH1*, *PTEN*, *GSTP1*, *FHIT*, *CDKN2A*, *DAPK1*, *MGMT*, *MLH1*, *RARB*, *RASSF1*, *SOCS1*, *TIMP3*, *VHL*, and *WIF1*. It also affects various histone modifications, including reducing methylation at H3K9, H3K27, H3K36, H3K79, and H3K4, as well as acetylation at H3 and H4. These changes help reactivate tumor suppressor genes and lead to reduced cell proliferation and migration [119].

Sun et al. found that chrysin inhibited the growth and proliferation of breast cancer cells (MDA-MB-231) in vitro. These results were supported by in vivo studies, where oral chrysin administration reduced tumor size and weight in a mouse. Chrysin was also identified as an HDAC8 inhibitor, suggesting that its anticancer effects are linked to hyperacetylation, increased p21 expression, decreased CTPS protein levels, and inhibited tumor growth [120].

In hepatocellular carcinoma, chrysin acts by interfering with RNA N6-methyladenosine (m6A) modification, an important epitranscriptomic mechanism that regulates RNA stability, splicing, and translation. Specifically, chrysin binds covalently to the amino acid residue threonine 205 of the enzyme enolase 1 (ENO1). ENO1 is known not only for its metabolic role in glycolysis but also for its function in stabilizing m6A-modified messenger RNAs that promote cancer progression.

By binding to ENO1, chrysin disrupts its ability to stabilize m6A-modified β-catenin mRNA. This leads to the degradation of β-catenin transcripts and a subsequent reduction in β-catenin protein levels. Since β-catenin is a key regulator of the Wnt/β-catenin signaling pathway, which drives cell proliferation, invasion, and metastasis in HCC, its downregulation suppresses these malignant behaviors. As a result, chrysin inhibits the epithelial–mesenchymal transition (EMT). Through this mechanism, chrysin effectively reduces the metastatic potential of HCC cells.

Animal studies further confirmed that chrysin treatment significantly suppressed tumor growth and metastasis in HCC models, without causing notable toxicity [121] (Table 3).

Chrysin also modulates miRNA expression, a key mechanism in its anticancer activity. In gastric cancer, it was shown that: increase let-7, miR-9, miR-22, miR-34, and miR-126, while decreasing miR-18a, miR-21, and miR-221. These miRNAs regulate a wide range of cellular targets. The let-7 family, commonly linked to tumor suppression, influences a broad spectrum of cellular pathways through the regulation of various genes (*BTG2*, *vesicular*, *integral membrane protein 1*, *VSNL1*; mitogen-activated protein kinase kinase kinase 4, *MAP4K4*; solute carrier family 35 member D2, *SLC35D2*; chromodomain helicase DNA binding protein 7, *CHD7*; Fraser Syndrome 1, *FRAS1*; ephrin receptor A4, *EPHA4*; phosphoglucomutase 2 like 1, *PGM2L1*). miR-18a regulates target involvement in hypoxia response (*HIF-1α*), DNA damage repair (*ATM*), and angiogenesis (estrogen receptor alpha: *ER-α*; vascular endothelial growth factor: *VEGF*). miR-21 is often considered an oncogene, and its targets are involved in cell survival and tumor suppression (*hMSH2*, *PTEN*, *Bcl-2*). miR-9 regulates targets that are involved in cell signaling and vesicular trafficking (growth factor receptor-bound protein 2, *GRB2*; *RAB34*). miR-34a is a tumor suppressor, and its targets are involved in cell proliferation and survival (platelet-derived growth factor receptor, *PDGFR*; mesenchymal–epithelial transition factor, *MET*). miR-22′s involvement in extracellular matrix remodeling, epithelial-to-mesenchymal transition (EMT), and cell cycle regulation (extracellular matrix, *ECM*; *MMP14*; *EMT*; estrogen receptor 1, *ESR1*; *SP1*; *HDAC4*; sirtuin 1, *SIRT1*; *p21*; *HIF-1a*; ecotropic viral integration site 1, *EVI1*; cyclin-dependent kinase 6, *CDK6*; ezrin, *EZR*; *Sp1*; *Wnt-1*). miR-221 is often associated with cancer, and its targets are involved in cell cycle control and tumor suppression (*PTEN*, *p27*, *p57*). miR-126 involved in angiogenesis and cell signaling (phosphoinositide-3-kinase regulatory subunit 2, *PI3KR2*; cytoplasmic receptor kinase, *Crk*; *VEGF-A*). These miRNA changes may contribute to chrysin’s anticancer effects by suppressing oncogenes, inhibiting proliferation and differentiation, migration, and invasion, reducing angiogenesis, and promoting apoptosis [122] (Table 4).

#### 2.3.4. Kaempferol

Kaempferol (Kae) is a naturally occurring flavonol found ubiquitously throughout the plant kingdom. It is present in many fruits and vegetables commonly consumed in the human diet, such as kale, spinach, broccoli, apples, grapes, and onions. Its widespread distribution also includes various medicinal herbs, highlighting its historical and contemporary significance [123]. Kaempferol has attracted considerable attention for its potential anticancer effects. Preclinical studies indicate that it may inhibit cancer cell proliferation, induce apoptosis in malignant cells, and impede metastasis, the spread of cancer to distant sites. Beyond its anticancer potential, research has also explored kaempferol’s involvement in other aspects of health. Kaempferol showed potential roles in cardiovascular health, neuroprotection, and diabetes management [124].

The study revealed that Kae modulates DNA methylation in bladder cancer, not by changing global 5-methylcytosine levels. Instead, Kae induces both hyper- and hypo-methylation at specific genomic locations, leading to differential gene expression. This leads to altered gene expression, including hypomethylation and upregulation of the WRN gene (a DNA repair gene), and hypermethylation and downregulation of replication factor C3 (RFC3), which promotes tumor growth [125,126]. Kaempferol was found to selectively inhibit DNMT3B expression without affecting DNMT1 or DNMT3A. This is significant because DNMT3B upregulation is often associated with the silencing of tumor suppressor genes in bladder cancer; its downregulation by Kae helps reactivate those genes and inhibit tumor progression. Kae promotes DNMT3B degradation via the ubiquitin-proteasome pathway, enhancing the ubiquitination of DNMT3B and its degradation. This process is likely mediated by Kae’s inhibition of the PI3K/AKT signaling pathway, which normally stabilizes DNMT3B [127,128].

In silico docking analyses demonstrated that kaempferol could effectively bind to the active sites of HDAC 2, 4, 7, and 8. Further in vitro profiling across all conserved human HDAC classes (I, II, and IV) confirmed these findings, revealing that kaempferol inhibited all tested HDACs. In hepatoma (HepG2, Hep3B) and colon (HCT-116) cancer cells, kaempferol increased histone H3 acetylation and significantly reduced cell viability and proliferation [129].

Kaempferol also downregulates histone methyltransferase G9a expression in gastric cancer cells in a dose-dependent manner, reducing the level of H3K9me2. Studies showed that G9a binds to the microtubule-associated protein 1B-light chain 3 (LC3B) promoter, a key protein in autophagy. However, kaempferol treatment, as well as G9a knockdown, diminishes G9a’s binding to this promoter, suggesting that kaempferol disrupts G9a’s regulatory influence on autophagy. This disruption is critical for kaempferol-induced autophagic cell death [130] (Table 3).

In colon cancer, kaempferol’s anticancer effects are partly due to its ability to increase miR-339-5p, which regulates alternative splicing of the pyruvate kinase M1/M2 (PKM) gene. Several miRNAs, including miR-339-5p, were identified as potential regulators of heterogeneous nuclear ribonucleoprotein A1 (hnRNPA1) and polypyrimidine tract binding protein 1 (PTBP1), two splicing factors crucial for PKM2 expression. Notably, kaempferol treatment led to a significant, dose-dependent increase in miR-339-5p expression. Overexpression of miR-339-5p decreased the expression of hnRNPA1, PTBP1, and PKM2, while increasing PKM1 expression. Luciferase reporter assays further validated that miR-339-5p directly targets the 3′-UTR of *hnRNPA1* and *PTBP1*, leading to their downregulation. Therefore, kaempferol’s upregulation of miR-339-5p ultimately results in decreased expression of these alternative splicing factors and, consequently, low expression of *PKM2*. Overexpression of miR-339-5p enhanced kaempferol’s ability to suppress cell proliferation and glycolysis, as evidenced by reduced glucose consumption, pyruvate acid accumulation, and ATP production. Conversely, inhibition of miR-339-5p reversed the suppression of *PKM2*, *PTBP1*, and *hnRNPA1* expression caused by kaempferol [131] (Table 4).

#### 2.3.5. Myricetin

Myricetin, a naturally occurring flavonoid found abundantly in various plant-based foods such as berries, fruits, vegetables, nuts, and tea. This compound is recognized for its potent antioxidant properties, anti-inflammatory effects, anti-diabetic and neuroprotective properties. Furthermore, research has explored its potential in cancer prevention and treatment, with studies indicating its ability to inhibit cancer cell growth and proliferation [132,133,134]. Myricetin inhibits histone lysine demethylases KDM4A-C by binding to their active sites, blocking the demethylation of H3K9me3 and H3K36me3. In prostate C4-2B cells, myricetin treatment led to an accumulation of H3K9me3 compared to untreated cells, with no corresponding change in H3K36me3. This indicates selective inhibition of H3K9me3 demethylation and consequent reduction in cell proliferation. These findings provide strong evidence for myricetin as a potent natural inhibitor of AR-positive prostate cancer cells [135] (Table 3).

#### 2.3.6. Fisetin

Fisetin is a naturally occurring flavonol, a type of plant pigment, found in various fruits and vegetables. Common dietary sources include strawberries, apples, grapes, onions, and cucumbers. Research indicates that fisetin possesses antioxidant, anti-inflammatory, and antitumor effects in various cell types and may exert its antitumor activity through a cancer-cell-specific mechanism [136].

Fisetin has been found to increase the expression of regulatory factor X associated protein (RFXAP) and histone demethylase KDM4A. This increase in KDM4A leads to a reduction in histone H3K36me, which in turn impairs homologous recombination repair in pancreatic ductal adenocarcinoma cells (PDACs). Since PDACs are known for their robust DNA repair capabilities, which contribute to their resistance to treatments. By hindering this excessive repair, fisetin induces DNA damage within the cancer cells, subsequently leading to S-phase arrest, effectively halting cancer cell proliferation. Furthermore, fisetin has been shown to enhance the sensitivity of PDACs to gemcitabine. These findings suggest that fisetin inhibits cancer cell proliferation by inducing DNA damage through RFXAP/KDM4A-dependent demethylation of histone H3K36 [137].

Fisetin treatment resulted in a significant reduction in genomic 5hmC levels within human renal cancer stem cells (HuRCSCs), accompanied by a decrease in both TET1 mRNA and protein expression. This indicates that fisetin effectively inhibits TET1 activity, thereby lowering 5-hmC levels. HuRCSCs, identified by the CD44+/CD105+ markers, were found to exhibit significantly higher levels of both genomic 5hmC and TET1 expression compared to non-stem renal cancer cells (HuNRCCs, CD44-/CD105-). Further investigation demonstrated that HuRCSCs also displayed higher expression of cyclin Y (CCNY) and CDK16, proteins crucial for cell cycle progression, compared to HuNRCCs. However, fisetin treatment led to a significant reduction in the expression of these proteins. The underlying mechanism involves TET1-mediated 5hmC modifications in the promoter regions of the *CCNY* and *CDK16* genes. By inhibiting TET1 activity, fisetin reduces these 5-hmC modifications, decreasing CCNY/CDK16 expression and disrupting cell cycle progression [137].

Fisetin regulates m6A RNA methylation to impair DNA damage repair in pancreatic ductal adenocarcinoma (PDAC). The compound induces DNA double-strand breaks (DSBs) and inhibits homologous recombination (HR) by targeting the m6A writer ZC3H13 and the chromatin remodeling subunit PHF10. Fisetin downregulates ZC3H13, reducing the m6A methylation of *PHF10* mRNA, which in turn decreases its translation through a YTHDF1-dependent mechanism. The loss of PHF10 leads to enhanced recruitment of γH2AX, RAD51, and 53BP1 at DSB sites and suppresses HR repair efficiency. Thus, fisetin enhances DNA damage and impairs HR repair via ZC3H13-mediated m6A modification of PHF10, providing a novel mechanism to sensitize PDACs to chemotherapy [138] (Table 3).

#### 2.3.7. Rhamnetin

Rhamnetin is a flavonoid found in plants like *Rhamnus petiolaris*, *Coriandrum sativum*, *Syzygium aromaticum*, and *Prunus cerasus* [139]. Specifically, studies have investigated its effects on melanogenesis and adipocyte differentiation and its potential role in radiosensitization [140,141]. As a nutraceutical flavonoid, rhamnetin has emerged as a promising candidate in the fight against ovarian cancer, particularly due to its interaction with HDAC2. In silico studies, including molecular docking and molecular dynamics simulations, provided compelling evidence of rhamnetin’s stable binding to the binding site cavity of the HDAC2 protein. In vitro assays confirmed that rhamnetin exerts cytotoxic effects by directly inhibiting HDAC2 activity. Furthermore, rhamnetin was shown to induce G1 phase cell cycle arrest, effectively halting the proliferation of ovarian cancer SKOV3 cells, and to trigger apoptosis [142] (Table 3).

Rhamnetin treatment increases the expression of miR-34a. Overexpression of miR-34a augments caspase-3 and caspase-9 activity, enhances p53 expression, and suppresses Notch-1 expression. This modulation of key signaling pathways increased apoptosis and decreased cell proliferation in MCF-7 breast cancer cells [143].

In non-small cell lung cancer (NSCLC) cells, rhamnetin significantly increases miR-34a expression, enhancing p53 activity and stability through increased acetylation. miR-34a also directly binds to the Notch-1 gene’s 3′-UTR, suppressing its expression. Rhamnetin and cirsiliol treatment effectively suppressed radiation-induced epithelial-to-mesenchymal transition, demonstrated by the elevation of the epithelial marker E-cadherin and the reduction in mesenchymal markers vimentin and fibronectin, observed at both mRNA and protein expression levels. Ultimately, by suppressing Notch-1, rhamnetin enhances NSCLC cell sensitivity to radiation therapy, leading to increased apoptosis and reduced cell proliferation and epithelial to mesenchymal transition [144].

Rhamnetin also effectively counteracts sorafenib resistance in hepatocellular carcinoma (HCC) by upregulating miR-148a expression. This upregulation of miR-148a is achieved through rhamnetin’s ability to enhance the acetylation of p53, which subsequently promotes p53′s recruitment to the miR-148a promoter region. miR-148a binds to the 3′-UTR of the *PXR* gene, leading to the suppression of pregnane X receptor (*PXR*), cytochrome P450 3A4 (*CYP3A4*), and P-glycoprotein (*P-GP*). This cascade reduces the metabolism and clearance of sorafenib, thereby enhancing its damaging effect on HCC cells. Rhamnetin, acting as a Sirt1 inhibitor, allows for increased p53 acetylation, thus promoting miR-148a expression. Clinical relevance is supported by findings that HCC patients with high miR-148a and low Sirt1 expression exhibit better prognoses when treated with sorafenib [145] (Table 4).

#### 2.3.8. Hesperidin

Hesperidin is a flavanone glycoside, a type of plant compound, primarily found in citrus fruits, especially oranges, lemons, and grapefruits [146]. Although research on hesperidin’s health effects is still limited, this flavonoid has demonstrated various biological and pharmacological properties, including anti-inflammatory, antioxidative, and lipid-lowering activities. Notably, studies have shown that hesperidin possesses anti-carcinogenic effects, inhibiting carcinogenesis in distinct cancer types like tongue, esophagus, and colon [147].

Hesperidin demonstrated a significant ability to induce hypomethylation of LINE-1 and ALU-M2 repetitive DNA sequences within HL60 leukemia cells in a concentration-dependent manner. In hepatocellular carcinoma, where aberrant DNA methylation patterns are common, hesperidin’s ability to modulate these patterns may contribute to its protective effects, suggesting chemopreventive potential [147].

Further supporting this potential, histopathological analysis demonstrates that exposure to diethylnitrosamine (DEN) and carbon tetrachloride (CCl4) in rats caused severe liver damage, including hydropic and fatty degeneration, fibrosis, clear cell foci, ultimately leading to hepatocellular carcinoma. DEN/CCl4 exposure caused significant hypermethylation of global DNA. However, hesperidin treatment reversed these effects by resulting in downregulation of microRNA-221 and fibroblast growth factor 2 (FGF-2), alongside the upregulation of miR-34a and Nrf2, Bcl-2, and caspase 3 genes, as well as global DNA hypomethylation. These findings highlight hesperidin’s chemopreventive and antiangiogenic effects, including inhibiting oncogene activity, promoting apoptosis, and protecting the liver from oxidative damage and inflammation, thereby ameliorating HCC progression [148].

In vitro results showed that hesperidin treatment increased miR-132 expression and decreased *ZEB2* expression in the non-small cell lung cancer tissues. Studies confirm that miR-132 directly targets and binds to the 3′-UTR of *ZEB2*, a transcription factor involved in epithelial–mesenchymal transition and cancer progression. By targeting ZEB2, hesperidin may inhibit tumor invasion and metastasis while promoting apoptosis [149].

Other studies showed that hesperidin significantly upregulated the expression of miR-34a in colon cancer cells A549 and H460. Additionally, hesperidin significantly increased the expression of p53, reduced the expression of EGFR, and downregulated the expression of PD-L1. Molecular mechanisms through which hesperidin promotes apoptosis in A549 and H460 lung cancer cells, focusing specifically on the miR-34a/PD-L1 signaling pathway [150]. Moreover, in breast cancer MCF-7 cells, hesperidin treatment led to the upregulation of miR-16 and miR-34a, while simultaneously downregulating miR-21. These microRNAs are known regulators of Bcl-2. These changes in miRNA expression inhibit Bcl-2 expression, thereby triggering apoptosis in MCF-7 cells [151] (Table 4).

#### 2.3.9. Naringenin

Naringenin (Nar) is a naturally occurring flavanone and is found predominantly in citrus fruits such as grapefruit, orange, and lemon, and exists in both aglycone and glycosidic forms. Naringenin exhibits a wide range of pharmacological properties, including antioxidant, anti-inflammatory, neuroprotective, anticancer, anti-atherosclerotic, and anti-diabetic activities. These effects are largely attributed to its chemical structure, particularly the presence of hydroxyl groups that react with reactive oxygen and nitrogen species [152].

Naringenin has been investigated for its anticancer effects against acute myeloid leukemia, specifically its impact on the XIST/miR-34a/HDAC1 pathway. Treatment with Nar resulted in the downregulation of XIST, Bcl-2, and HDAC1, and the upregulation of miR-34a and caspase-3. It was found that XIST and HDAC1 interact with miR-34a, and that overexpression of miR-34a induces apoptosis by downregulating Bcl-2 and upregulating caspase-3, while simultaneously reducing XIST and HDAC1 expression [153] (Table 4).

#### 2.3.10. Silibinin

Silibinin, a prominent flavonolignan, is a key active component derived from silymarin, the standardized extract of milk thistle seeds (*Silybum marianum*) [154]. As the primary active component of silymarin, silibinin exists as a mixture of two diastereomers—silybin A and silybin B—which contribute to its multifaceted biological activities. Silibinin functions as an antioxidant, anti-inflammatory, anti-proliferative, pro-apoptotic, anti-metastatic, and anti-angiogenic agent. Research has demonstrated that silibinin exhibits significant anticancer activity against a broad spectrum of human cancers, including those of the liver, lungs, breast, prostate, colorectal, skin, and bladder [155].

Silibinin treatment of DU145 and PC3 prostate cancer cell lines resulted in a significant reduction in the expression of PRC2 complex members, including EZH2, SUZ12, and EED. Interestingly, while EZH2 protein levels decreased, a modest increase in H3K27me3 was observed. Silibinin treatment led to a concentration-dependent decrease in phosphorylation of AKT (pAKT, Ser473), which subsequently reduced phosphorylation of EZH2 (pEZH2, Ser21). The decrease in pAkt and pEZH2 phosphorylation by silibinin allowed for increased H3K27me3 levels despite the reduction in EZH2 protein. Furthermore, silibinin caused a modest, concentration-dependent increase in overall DNA methyltransferase activity, a decrease in HDAC1 and HDAC2 expression levels, and was associated with EMT and cancer stem cell phenotypes in prostate cancer [156]. Notably, HDAC inhibitors alone did not induce cell death in these cells, highlighting the synergistic effect of silibinin. In colorectal cancer cells SW480 and SW620, silibinin also caused a significantly reduced DNMT level, but did not alter HDAC activity. However, treatment with silibinin in combination with two HDAC inhibitors (SAHA and TSA) led to increased cell death in both colorectal cancer cells [157]. A similar synergic effect was observed in non-small cell lung cancer H1299, where silibinin enhanced the effects of both the histone deacetylase inhibitor (TSA) and DNA methyltransferase inhibitor (AZA), restoring E-cadherin expression, downregulating Zeb1, and inhibiting the aggressive behavior of these cancer cells [158].

Studies also demonstrated that silibinin-mediated inhibition of HuH7 xenograft growth coincided with a significant elevation of H3ac and H4ac, thus confirming silibinin’s in vivo effects on histone acetylation and its potential role in suppressing HCC growth [159].

Moreover, silibinin decreased the presence of H3K4me3 and H3ac at the KRAS promoter region in bladder cancer cells. This reduction in activating histone marks suggests that silibinin epigenetically represses KRAS gene transcription, ultimately leading to the observed inhibition of cancer cell proliferation and invasion [160] (Table 3).

Silibinin was shown to significantly reduce the expression of oncogenic miRs, miR-21 and miR-155, in breast cancer. In silico analysis identified potential target genes of these miRs within the intrinsic and extrinsic apoptosis pathways, including CASP-9, BID, APAF-1, and p53. The study proposes that silibinin’s anticancer effects are mediated through multiple mechanisms, including the suppression of miR-21 and miR-155, leading to the upregulation of CASP-9, p53, and BID [161] (Table 3).

In breast cancer cell lines MCF-7 and T47D, silibinin consistently downregulated miR-21 expression. Consequently, the reduction in miR-21 expression effectively alleviates this suppression, enabling the upregulation of crucial tumor-suppressing and apoptosis-inducing proteins (*PDCD4*, *PTEN*, *FASL*, *CASP* genes, and p53), resulting in apoptosis, cell proliferation inhibition, and overall tumor growth suppression [162] (Table 3).

Silibinin encapsulated in Polymersome Nanoparticles (SPNs) significantly upregulated miR-34a and downregulated the oncogenic miRNAs, miR-221 and miR-222, in colorectal cancer cell HT-29. Bioinformatics and quantitative analyses confirmed that miR-34a targets several apoptotic genes, including *TP53*, *BAX*, *CASP9*, *CASP3*, and *CASP8*. SPN’s treatment led to upregulation of these genes, activating both intrinsic and extrinsic apoptotic pathways. Additionally, Bcl-2 was downregulated, contributing to further apoptosis [163] (Table 3).

Silibinin has also shown potential in overcoming erlotinib resistance in non-small cell lung cancer tumors. Erlotinib-resistant tumors displayed an altered miRNA expression pattern; increased miR-21 and decreased miR-200c, both of which are associated with EMT. Silibinin reversed this pattern, downregulating miR-21 and upregulating miR-200c, effectively restoring the miRNA profile to that of erlotinib-sensitive tumors. This miRNA modulation correlated with a reduction in the expression of mesenchymal markers like SNAIL1, ZEB1, ZEB2, and N-cadherin, indicating a reversal of EMT. In vitro studies confirmed that silibinin could reverse the mesenchymal phenotype of PC-9/Erl-R cells, restoring an epithelial morphology and reducing cell migration [164].

Silibinin treatment led to the downregulation of miR-20b, an miRNA associated with breast cancer progression, and a concurrent upregulation of key apoptotic genes, including *BCL2L11* (*BIM*), *PTEN*, and *caspase 9*. Computational docking analyses further revealed strong interactions between silibinin and miR-20b, suggesting a direct inhibitory effect on this miRNA, and also a strong interaction between silibinin and Erbb2. The study showed that silibinin exerts its antitumorigenic activity through a combination of mechanisms. These include the downregulation of miR-20b, the upregulation of BCL2L11, PTEN, and caspase 9, which activate critical apoptotic pathways, and the interaction with Erbb2. These combined effects culminate in apoptosis induction, cell cycle arrest, and the overall inhibition of cancer cell growth [165].

Additionally, silibinin significantly enhanced the efficacy of miR-133a in breast cancer cells. Under silibinin treatment, miR-133a effectively reduces the expression of *EGFR* and *PIK3C2A*, key components of the PAM pathway, resulting in decreased tumor cell growth and development [166]. The study revealed a significant decrease in miR-181a expression in silibinin-treated SK-BR-3 cells. Reduced expression of miR-181a directly contributes to the observed inhibition of cell growth and induction of apoptosis [167] (Table 4).

#### 2.3.11. Genistein

Genistein, a naturally occurring isoflavone predominantly found in soybeans and soy-based products, is classified as a phytoestrogen due to its structural similarity to estrogen. Clinical trials explored genistein’s potential benefits as an antioxidant, anticancer, cytotoxic, and anti-inflammatory agent, as well as its therapeutic effects on climacteric symptoms, diabetes, lipid metabolism, depression, neurodegeneration, bone health, and cardiovascular disease [168].

In neuroblastoma (NB) cells, genistein treatment led to demethylation of the *CDH5* promoter and decreased the expression of DNMT3B. In vivo, genistein inhibited NB growth by increasing CDH5 protein expression by reducing the *CDH5* gene methylation [169].

Furthermore, genistein was found to inhibit DNA methyltransferase activity, particularly DNMT1, in prostate cancer cells. The compound also decreased the binding of MBD2, a protein that binds to methylated DNA and represses gene transcription. Notably, genistein induced active histone modifications at the *BTG3* transcription start site, including an increase in histones H3ac and H4ac, H3K4me2, and H3K4me3. These epigenetic changes resulted in a significant increase in *BTG3* mRNA levels. BTG3, a negative regulator of the cell cycle and a potential tumor suppressor, is silenced by hypermethylation in prostate cancer, and its reactivation by genistein may inhibit cancer cell proliferation [170].

In prostate cancer cells LNCaP and DuPro, genistein treatment led to significant chromatin remodeling, characterized by an enrichment of H3ac, H4ac, and H3K4me2, near the transcription start sites of p16 and p21. Researchers also found that genistein significantly increased the mRNA expression of histone acetyltransferases, including *p300*, *p300/CBP-associated factor* (*PCAF*), *CREB binding protein* (*CREBBP*), and *HAT1*. These findings collectively suggest that genistein upregulates p16 and p21 expression through an epigenetic mechanism involving chromatin remodeling and increased HAT activity, independent of DNA methylation, ultimately inhibiting cell proliferation [171].

Synergistic effects of genistein and HDAC inhibitor Trichostin (TSA) observed in lung cancer cells. The study demonstrated that genistein significantly enhanced the growth-inhibitory and apoptosis-inducing effects of TSA in A549 and H460 lung cancer cells. TSA alone increased histone H3/H4 acetylation and p53 acetylation, and genistein significantly augmented this effect. TSA combined with genistein significantly increased p300 expression and HAT activity in both cell lines. Genistein alone also showed a dose-dependent increase in p300 expression and HAT activity in A549 cells [172].

In breast cancer cells, genistein was found to directly bind the active site of DNMT1, inhibiting its activity. This led to demethylation of promoter regions in several genes, including *ATM*, *APC*, and *PTEN*, thereby reducing cell viability [173].

In HeLa cervical cancer cells, genistein significantly downregulated a wide range of chromatin-modifying enzymes, including DNMT1, DNMT3A, DNMT3B, HDAC1, HDAC5, HDAC6, and upregulated several other chromatin modifiers, such as SETD5, SETD7, SETD6, CIITA, and ESCO2. Genistein inhibited the enzymatic activity of DNA methyltransferases, histone deacetylases, and histone methyltransferase H3K9. It also induced a time-dependent decrease in global DNA methylation and significantly reduced the 5′ CpG methylation of several TSG promoters, including *APC*, *DAPK1*, *FHIT*, *PTEN*, *GSTP1*, *RARB*, *RASSF1*, *CDH1*, *MLH1*, *SOC51*, *TIMP3*, *CDH13*, *MGMT*, *VHL*. Genistein regulates the expression of these genes, thereby inhibiting cancer cell proliferation, migration, and angiogenesis, while inducing apoptosis [174].

A study showed that long-term genistein treatment can induce epigenetic changes in breast cancer cells MCF-7. It resulted in a reduced basal growth rate and rendered the cells unresponsive to estradiol and epidermal growth factor. These cells exhibited depleted levels of H3ac, sustained increased levels of pro-caspase 9, and reduced levels of cyclin D1, leading to inhibited cell proliferation and increased apoptosis [175].

In SW480 and HCT15 colon cancer cell lines, genistein treatment significantly increased histone H3 acetylation at both the promoter and coding regions of the *DKK1* gene. This epigenetic modification was associated with enhanced RNA polymerase II binding at the DKK1 promoter region, suggesting enhanced transcriptional activity. This mechanism contributes to the inhibition of the WNT/β-catenin pathway, cell cycle arrest, and reduced proliferation [176] (Table 3).

Additionally, genistein treatment significantly upregulated miR-27a expression in A549 human lung cancer cells. This upregulation led to a decrease in MET, resulting in the inhibition of non-small cell lung cancer (NSCLC) proliferation [177] (Table 4).

#### 2.3.12. Daidzein

Daidzein (DPN), a naturally occurring isoflavone, is predominantly found in soybeans and soy-derived products, making soy-based foods the primary dietary source. This includes common soy products such as tofu, soy milk, tempeh, miso, and soy flour. Another notable source is the root of the kudzu plant (*Pueraria lobata*), which has been traditionally utilized in Chinese medicine [178,179]. Extensive research has explored its pharmacological properties, revealing its role in cancer prevention, cardiovascular diseases, diabetes management, osteoporosis treatment, skin protection, and neurodegenerative disease mitigation [180].

The study showed that daidzein could regulate DNA methylation. Specifically, it significantly decreased methylation at the promoter regions of *BRCA1*, *GSTP1*, and Eph receptor B2 (*EPHB2*) in DU-145 and PC-3 prostate cancer cell lines, leading to cell cycle arrest in the G0/G1 phase [181]. Further research indicated that daidzein exerts a protective effect against breast cancer by influencing DNA methylation and the expression of *BRCA1* and *BRCA2* oncogene suppressor genes. Daidzein reversed hypermethylation within their promoter regions, which are commonly altered during cancer development [182].

In both MCF7 and MDA-MB-231 breast cancer cell lines, daidzein significantly decreased the levels of repressive histone marks H3K9me3 and H3K27me3, while increasing histone acetylation marks H4K8ac and H3K4ac. Immunohistochemical staining showed increased p300 expression (associated with H4K8ac) and decreased EZH2 expression (linked to H3K27me3) after daidzein treatment. The study concluded that daidzein is involved in the regulation of cancer progression genes (*EZH2*, *BRCA1*, *ERα*, *ERβ*, *SRC3*, *and p300*) through regulating its histone methylation and acetylation and decreasing proliferation, migration, and invasion [183] (Table 3).

Another study demonstrated that a combination of 5-aza-2′-deoxycytidine (AzaC) and daizein significantly reduced the viability of glioblastoma U87MG and LN18 cells. While AzaC effectively demethylates the hypermethylated promoter region of the tumor suppressor miR-137 gene, daizein alone did not exhibit this demethylating effect. However, miR-137 mimic transfection combined with daidzein treatment inhibited glioblastoma cell invasion and most effectively suppressed the expression of survival, angiogenic, growth, and invasion-related factors, including AKT, NF-κB, VEGF, b-FGF, EGFR, MMP-9, and MMP-2. Furthermore, the combined treatment significantly inhibits in vitro angiogenic network formation and VEGF expression, indicating a potent anti-angiogenic effect [184] (Table 4).

#### 2.3.13. Delphinidin

Delphinidin (Dp), a vibrant purple pigment from the anthocyanidin family of water-soluble flavonoids, is abundantly present in various fruits and vegetables such as berries, eggplant, and grapes, contributing to their distinctive coloration. Cancer research has shown that delphinidin possesses promising anticancer properties, including the induction of apoptosis in cancer cells and the inhibition of cell proliferation and angiogenesis [185].

The study showed that delphinidin exerts protective effects against skin cancer. It demonstrated that delphinidin significantly inhibits TPA-induced neoplastic cell transformation in mouse epidermal JB6 P+ cells, an effect attributed to the activation of the Nrf2-ARE pathway. Delphinidin enhances this pathway, evidenced by increased ARE-driven luciferase activity and elevated mRNA and protein expression of Nrf2 downstream genes, such as heme oxygenase-1 (HO-1). Furthermore, the study revealed that delphinidin induces demethylation of 15 CpG sites in the mouse Nrf2 promoter region. This epigenetic modification correlated with decreased protein expression of DNA methyltransferases and histone deacetylases, indicating that delphinidin acts as an epigenetic demethylating agent. By inhibiting these enzymes, delphinidin reduces methylation on the Nrf2 promoter, allowing for increased expression of Nrf2 and its downstream antioxidant genes [186] (Table 3).

Another study demonstrated that delphinidin treatment significantly upregulates the expression of miR-34a in breast cancer tissues and cells. Importantly, delphinidin exerts this effect by inhibiting HOTAIR, an oncogene that plays a key role in breast cancer progression. Overexpression of HOTAIR effectively blocks delphinidin’s ability to upregulate miR-34a, highlighting that this effect is mediated via HOTAIR inhibition. Delphinidin also exhibits epigenetic regulatory effects by decreasing the binding of HOTAIR to the miR-34a promoter and reducing the occupancy of EZH2 and H3K27me3 at the same promoter. The study further shows that delphinidin effectively suppresses the Wnt/β-catenin signaling pathway through modulation of miR-34a and HOTAIR. Specifically, delphinidin treatment significantly reduces the expression of key proteins involved in this pathway, including β-catenin, phosphorylated glycogen synthase kinase 3 beta (GSK-3β), c-Myc, cyclin-D1, and MMP-7, all of which are critical for cell proliferation, survival, and metastasis [187].

Additional studies indicate that delphinidin inhibits the migration and invasion of colorectal cancer cells by upregulating miR-204-3p, which in turn suppresses the αV/αV/β3-integrin/FAK axis and inhibits the focal adhesion kinase FAK/Src/paxillin signaling pathway, which are crucial for cell adhesion and motility. Moreover, delphinidin disrupts cytoskeletal organization and suppresses the epithelial–mesenchymal transition process [188] (Table 4).

#### 2.3.14. Resveratrol

Resveratrol (RSV), a naturally occurring stilbenoid, is found in grapes, blueberries, raspberries, mulberries, and peanuts. It modulates various signaling pathways, effectively limiting tumor cell spread, protecting nerve cells from damage, aiding in diabetes prevention, and generally exhibiting anti-aging effects. RSV has demonstrated beneficial effects across a broad spectrum of pathological conditions, including neoplastic diseases, neurodegeneration, metabolic syndrome, diabetes, obesity, cardiovascular diseases, immune disorders, and bacterial, viral, and fungal infections [189,190].

Resveratrol exhibits significant anticancer effects in breast cancer cells through epigenetic modifications, primarily by inducing hypermethylation of genes associated with key oncogenic signaling pathways. Research has shown that RSV-hypermethylated targets are enriched in oncogenic signal transduction pathways, such as Notch, Wnt, Hedgehog, TGF-β, MAPK, AKT, GLI family zinc finger 2 (GLI2), and WNT4. Silencing of GLI2 and WNT4 leads to the downregulation of downstream target genes of both the Hedgehog and Wnt pathways, including Bcl2, epithelial cell adhesion molecule (EpCam), CCND1, and cysteine-rich protein 61 (CYR61), indicating comprehensive inhibition of these oncogenic signals. Furthermore, RSV induces H3K27me3 and decreases H3K27ac and H3K9ac, at the GLI2 and WNT4 enhancer regions. This leads to a more condensed chromatin structure, reducing transcription factors’ accessibility, particularly OCT1, resulting in decreased proliferation, metastasis, and increased apoptosis [191].

Another study revealed that resveratrol significantly alters the methylation status of numerous genes in MDA-MB-231 breast cancer cells, inducing both hypermethylation and hypomethylation. Notably, RSV treatment led to hypomethylation of several key tumor suppressor genes, including insulin growth receptor 2R (*IGF2R*), *TP53*, *FOXO3*, SRY (Sex-determining Region Y-Box 17) (*SOX17*), *SLIT3*, and Cysteine Dioxygenase 1 (*CDO1*) and hypermethylation of oncogenes, such as *AURKA*, cyclin B1 (*CCNB1*), and heksokinase 2 (*HK2*). These findings demonstrate RSV’s potential to modulate key pathways critical to cancer development and progression [192].

RSV treatment also reduces the expression of methyltransferases PRMT5 and EZH2, at both the transcriptional and translational levels in MCF-7 and MDA-MB-231 cells. This correlates with a reduction in repressive histone methylation marks, H4R3me2 and H3K27me3. Concurrently, RSV modulates histone acetylation by decreasing the activity and expression of HDAC1 while increasing the activity and expression of histone acetyltransferases KAT2A and KAT3B. This dual action results in an increase in activating histone acetylation marks, H3K9ac and H3K27ac. Chromatin immunoprecipitation (ChIP) assays provide compelling evidence that RSV enhances activating histone acetylation and reduces repressive methylation at the promoters of *BRCA1*, *p53*, and p21, restoring their expression and inhibiting the growth and proliferation of breast cancer cells [193].

In silico docking analyses demonstrated that RSV can effectively bind to the active sites of class I and II HDAC enzymes, interacting with crucial residues, including the catalytic zinc ion. This HDAC inhibition correlated with significant antiproliferative effects in HepG2 hepatoma cells, marked by hyperacetylation of histone H3. Notably, resveratrol showed no cytotoxic side effects on non-malignant primary human hepatocytes in vitro [194] (Table 3).

Furthermore, resveratrol treatment upregulates miR-15a and miR-16-1 expression in acute lymphoblastic leukemia CCRF-CEM cells, mirroring the time- and dose-dependent pattern observed in apoptosis induction. These miRNAs, known to be downregulated in certain leukemias and to play a crucial role in apoptosis regulation by targeting the anti-apoptotic gene Bcl2, are believed to contribute significantly to the resveratrol-induced cell death [195].

Research also showed that resveratrol significantly decreases the expression of miR-21 in bladder cancer cells. It was shown that downregulation of miR-21 increased apoptosis and caspase-3 activity, while overexpression of miR-21 reversed the resveratrol-induced increase in caspase-3 activity. The inhibition of miR-21 also leads to reduced AKT activity, subsequently decreasing Bcl-2 expression and promoting apoptosis [196].

It was also shown that a combination of miR-150 and RSV led to an increase in MDM2 expression but further decreased RUNX3 and RB expression. Resveratrol alone also decreased MDM2 and RB expression. These alterations in gene expression, coupled with the observed increase in apoptosis, suggest that both miR-150 and resveratrol, particularly in combination, exert potent anticancer effects in HL-60 cells [197] (Table 4).

#### 2.3.15. Pterostilbene

Pterostilbene (PTS), a naturally occurring stilbenoid, is found in blueberries, grapes, and heartwood demonstrated potent antioxidant activity in both in vitro and in vivo models. These antioxidant properties contribute to its preventative and therapeutic potential across various diseases including cancer, neurological disorders, inflammation, vascular diseases, and diabetes [198].

PTS influences the interaction between the oncogenic transcription factor *OCT1* and the de novo DNA methyltransferase DNMT3B in invasive breast cancer cells. Reduced *OCT1* and increased DNMT3B binding are predominantly associated with oncogenic processes such as cell growth, cell cycle regulation, adhesion, apoptosis, and cell motility. PTS treatment facilitates DNMT3B recruitment to regions with diminished *OCT1* binding, resulting in hypermethylation of the protein kinase C alpha (*PRKCA*) promoter and the DNA-damage-inducible transcript 2 (*DANT2*) and troponin T2 (*TNNT2*) enhancers, thereby inhibiting cancer cell growth [199].

Research also highlighted the synergistic potential of combining resveratrol and pterostilbene in the treatment of triple-negative breast cancer, particularly through epigenetic and cellular mechanisms. The combination inhibits SIRT1, and this effect is linked to decreased γ-H2AX, hTERT, and telomerase activity, suggesting impaired DNA repair. Thus, the combination effectively targets TNBC cells through multiple mechanisms, including apoptosis induction, cell cycle arrest, SIRT1 inhibition, and modulation of DNA damage response [200].

Further studies demonstrated that pterostilbene downregulates the expression of MTA1, a component of the MTA1/HDAC1/NuRD deacetylation complex. This downregulation destabilizes the interactions within this complex, leading to a reduction in HDAC1 activity. This leads to increased acetylated PTEN levels, enhancing its tumor suppressor activity. Additionally, pterostilbene directly downregulates HDAC enzymes, disrupting the balance between histone acetyltransferase HAT and HDAC activities. This imbalance favors HAT-induced acetylation, further contributing to the overall increase in histone acetylation. The increased PTEN acetylation, in turn, contributes to decreased p-AKT activation. These molecular changes ultimately result in the repression of hepatocellular carcinoma cell growth, inhibition of cell invasion, and induction of apoptosis [201] (Table 3).

Studies also showed that PTS enhances prostate cancer cells’ susceptibility to natural killer cell-mediated cytotoxicity, via miR-20a. Researchers found that treating PC3 and DU145 prostate cancer cells with PTS notably enhanced their susceptibility to human natural killer NK92 cell-mediated killing. The increased cytotoxicity was shown to rely on natural killer group 2 member D activation (NKG2D), as it was effectively inhibited by anti-NKG2D monoclonal antibodies. Interestingly, PTS also resulted in elevated intracellular levels of interferon-gamma (IFN-γ), CD107a (a marker for NK cell degranulation) in NK92 cells (a human natural killer cell line), suggesting enhanced NK cell activity. Research revealed that PTS boosts the susceptibility of prostate cancer cells to NK cell-mediated cytotoxicity by upregulating MICA/B (MHC Class I Chain-Related Proteins A and B) expression, which are ligands for NKG2D, and reducing TGF-β1 secretion through the inhibition of miR-20a [202].

PTS significantly downregulates oncogenic miRs, including miRs-17, -20a, -106a, and -106b in prostate cancer. These miRNAs normally suppress PTEN expression by binding to the PTEN 3′-UTR. In vivo xenograft models confirmed that PTS decreases tumor growth in cells overexpressing these miRNAs, enhancing PTEN expression and increasing tumor cell apoptosis [203].

In hepatocellular carcinoma SMMC-7721 cells, PTS was shown to downregulate miR-19a expression, leading to increased PTEN expression, and inhibiting the PI3K/AKT signaling pathway. This results in reduced cell viability, cell cycle arrest, increased apoptosis, and decreased invasion [204]. In endometrial carcinoma, PTS downregulates miR-663b, which targets the 3′-UTR of the pro-apoptotic gene BCL2L14. Through this pathway, PTS induces apoptosis [205] (Table 4).

#### 2.3.16. Piceatannol

Piceatannol, a naturally occurring polyphenolic stilbene, is found in grapes, red wine, passion fruit, white tea, and Japanese knotweed [206]. This compound exhibits a wide range of anticancer activities, making it a compound of significant research interest [207].

Microarray analysis demonstrated that piceatannol treatment downregulates miR-21, a key oncogenic microRNA in osteosarcoma. PTEN, identified as a direct target of miR-21, is upregulated following piceatannol treatment, resulting in downregulation of phosphorylated AKT. This suggests that piceatannol blocks the PTEN/AKT signaling pathway and attenuates tumor growth while inducing apoptosis [208].

In colorectal cancer cell lines HCT-116 and HT-29, piceatannol significantly upregulated miR-129, leading to reduced Bcl-2 and increased Bax expression. This shift in balance triggered caspase-3 activation and apoptosis [209]. Similar effects were observed in pancreatic cancer cells (SW1990 and PANC-1), where piceatannol-induced apoptosis was mediated through the upregulation of miR-125b [210], and in melanoma cells through miR-181a upregulation [211] (Table 4).

#### 2.3.17. Gallic Acid

Gallic acid (GA) is a ubiquitous phenolic acid found in guava, grapes, pomegranate, avocado, blackcurrant, and mango [212]. Studies have highlighted its potential in the treatment of infections, inflammation, cardiovascular diseases, gastrointestinal disorders, metabolic syndromes, and neurological conditions, as well as its anticancer properties [213].

The study revealed that GA treatment significantly altered genomic 5-methyl-2-deoxycytidine (5-mC) content in lung and oral cancer cell lines. Furthermore, GA significantly reduced both nuclear and cytoplasmic levels of DNMT1, particularly in H1299 cells, and also impacted DNMT3B and DNMT3A levels, with long-term treatment leading to decreased nuclear DNMT3B. Additionally, GA treatment upregulated several genes, including cyclin E (*CCNE2*), cyclin D3 (*CCND3*), *cyclin-dependent kinase inhibitor 1A* (*CDKN1A*), and cyclin B1 (*CCNB1*), which are critical components of the growth arrest and DNA damage-inducible 45 (GADD45) signaling pathway, cell cycle regulation, and cell cycle checkpoint control. This gene reactivation, attributed to promoter demethylation, contributed to the inhibition of cancer cell growth [214] (Table 3).

Another analysis revealed that GA treatment significantly upregulated miR-518b in chondrosarcoma SW1353 cells. GA induces apoptosis in SW1353 via a mitochondria-dependent pathway through decreased Bcl-2 and increased Bax expression, along with enhanced activation of caspase-3 and caspase-9 [215]. In colorectal cancer cells, GA upregulated miR-1247-3p, leading to a reduction in integrin αV/β3 and inhibition of paxillin activation. In vivo experiments using a xenograft model demonstrated that GA administration effectively suppressed tumor growth and liver metastasis originating from DLD-1 cells. These findings suggest that GA inhibits CRC metastasis through the suppression of the integrin/FAK axis, a process mediated by the upregulation of miR-1247-3p [216].

GA exhibits a dual nature, with low concentrations offering protective effects, while higher concentrations induce cytotoxicity in glioblastoma T98G cells. Specifically, at lower concentrations (≤25 μg/mL), GA reduced the expression of miR-17-3p, miR-21-5p, and miR-421-5p, whereas higher concentrations (>25 μg/mL) increased their expression. This modulation of miRNA expression directly correlated with GA’s impact on cell proliferation and survival. High doses of gallic acid lead to a reduction in mitochondrial antioxidant capacity (via elevated miR-17), a suppression of cell growth (through increased miR-21), and a compromised ability to repair cellular damage (increased miR-421) [217] (Table 4).

#### 2.3.18. Ellagic Acid

Ellagic acid (EA), a naturally occurring polyphenol antioxidant, is abundant in various fruits and nuts, notably pomegranates, berries (such as raspberries and strawberries), and walnuts [218]. It has shown promising anticancer potential, with studies indicating its ability to inhibit tumor growth, prevent cancer cell spread, and induce apoptosis across various cancer types, including breast, colon, prostate, and lung cancer [219,220].

**Figure 5:**
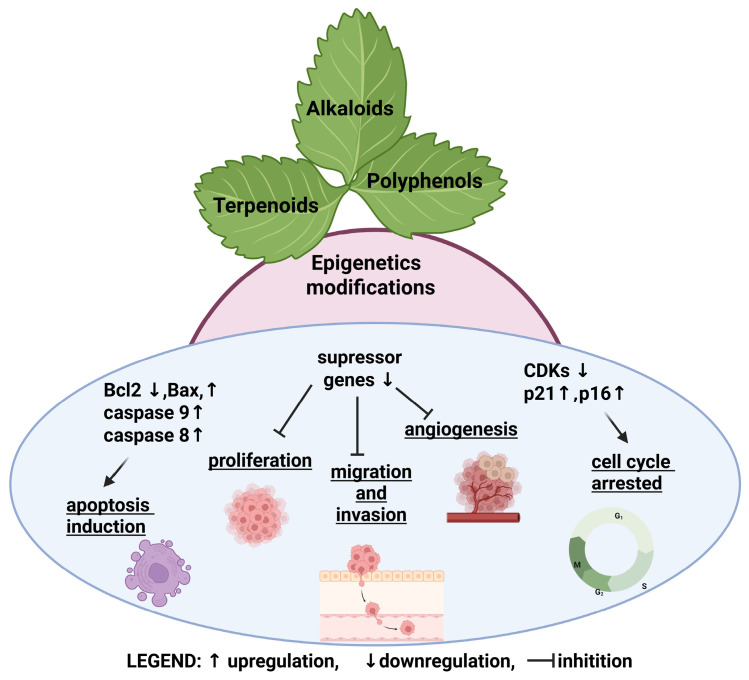
**Comparative mechanisms of epigenetic regulation by alkaloids, terpenoids, and polyphenols in cancer cells.** This figure illustrates the shared and distinct epigenetic mechanisms through which alkaloids, terpenoids, and polyphenols modulate key cellular processes in cancer. Epigenetic modifications, these natural compounds can regulate apoptosis, cell cycle progression, and the expression of tumor suppressor genes. Such epigenetic modulation affects multiple aspects of tumor progression, including cell proliferation, migration, invasion, and angiogenesis, highlighting the potential of these compounds as metabolic-epigenetic modulators and therapeutic agents in cancer prevention and treatment. Legend: ↑ upregulation, ↓ downregulation, ┴ inhibition.

EA treatment significantly reduced H3K27me3 and H4R3me2 catalytic products of EZH2 and PRMT5, respectively, in vitro and in vivo, indicating effective inhibition of these enzymes’ activities. In vitro findings were validated by in vivo studies using a tumor xenograft mouse model, which showed a notable reduction in tumor volume following EA treatment. This was accompanied by decreased levels of H3K27me3, H4R3me2, and the proliferation marker Ki-67 [221] (Figure 5).

Additionally, EA treatment upregulated miR-122 expression in patients with hepatocellular carcinoma. These patients also showed downregulated expression of EMT markers (vimentin, TGF-β1, FSCN1, MMP-9, VEGF) [222] (Figure 4).

## 3. Bioavailability Challenges and Therapeutic Duality of Natural Compounds: Strategies for Enhancement

Natural compounds, such as alkaloids, terpenes, and polyphenols, have garnered significant attention due to their therapeutic potential. However, their clinical applicability is often limited by challenges related to bioavailability and pharmacokinetics. A major issue is poor water solubility, which restricts absorption, while extensive metabolism in the liver and intestines further reduces the amount of active compound reaching systemic circulation [223,224,225,226]. Microbial metabolism can also transform natural compounds into various metabolites, affecting their bioactivity [227]. Interactions with tannins and dietary fiber may further limit absorption by forming complexes with these compounds [228,229,230].

The bioavailability of alkaloids is strongly dependent on their chemical structure, particularly lipophilicity and pKa, and their absorption is influenced by pH, being more efficient under alkaline conditions [231,232]. In contrast, terpenoids are highly lipophilic, which results in low water solubility, volatility, and chemical instability, limiting gastrointestinal absorption [233]. Polyphenols, on the other hand, are poorly soluble, undergo extensive metabolism, and can be actively transported back into the intestinal lumen by membrane transporters such as P-gp, MRP2, and BCRP [234,235].

Many studies have highlighted that the biological and epigenetic effects of natural compounds are often dose- or concentration-dependent, exhibiting biphasic behavior. At low concentrations, these compounds generally exert cytoprotective, antioxidant, and epigenetically modulatory effects, whereas at higher doses they may become cytotoxic, inducing apoptosis or oxidative stress in cells with different characteristics [236,237,238]. This dual nature is particularly evident among alkaloids, such as sanguinarine, evodiamine, and vincristine, which can promote DNA damage and apoptosis in tumor cells, while preserving genomic stability in normal tissues [239,240,241]. Similarly, terpenes, including betulinic acid, ursolic acid, and lycopene, exhibit protective antioxidant and histone-modulating properties at physiological levels, but display pro-oxidant and cytotoxic effects at pharmacological concentrations [242]. Polyphenols, such as resveratrol, genistein, and quercetin, show dose-specific dual roles, acting as epigenetic modulators of DNMTs and HDACs at low doses, while triggering pro-apoptotic signaling under higher exposure [243,244,245].

To improve their therapeutic efficacy, research has explored multiple strategies to enhance bioavailability, including chemical modification (e.g., introducing functional groups, forming esters, salts, or glycosides) to improve solubility and stability [246], lipid-based formulations such as SLN, liposomes, nanoemulsions, or microcapsules [247,248], encapsulation in biocompatible polymers to prolong half-life and protect from degradation, and co-administration with bioenhancers, such as piperine, which inhibit metabolic enzymes (e.g., CYP3A4, CYP2C9) and efflux transporters (e.g., P-gp), to increase systemic concentrations [249,250].

## 4. Clinical Applications

The exploration of natural compounds in clinical trials represents a significant and evolving area of medical research, rooted in the long-standing use of plants and other natural sources in traditional healing practices [251,252]. This resurgence of interest is fueled by the desire to discover safer, more effective treatments, particularly in light of the potential toxicities associated with synthetic pharmaceuticals. Natural compounds, due to their diverse chemical structures and biological activities, offer a rich source of potential therapeutic agents [253]. However, their integration into modern medicine necessitates rigorous scientific evaluation through the established clinical trial process.

This process, which includes Phase I, II, and III trials, ensures that natural compounds meet the same standards of safety and efficacy as synthetic drugs. Despite their potential, natural compounds present unique challenges. Standardization of extracts, ensuring consistent quality and composition, can be difficult. Similarly, bioavailability issues, such as poor absorption or rapid metabolism, can limit their therapeutic effectiveness. Potential drug interactions with existing medications also require careful consideration [254]. Strict adherence to regulatory requirements is crucial for the successful commercialization of natural compound-based therapies.

Current research focuses on various therapeutic areas, including cancer [255], cardiovascular diseases [256], neurological disorders [257], diabetes [258], and inflammatory conditions [259]. Organizations like the National Center for Complementary and Integrative Health (NCCIH) play a vital role in supporting and guiding research efforts in this field. Ultimately, the successful translation of natural compounds into clinical practice relies on robust scientific investigation, emphasizing the need for well-designed clinical trials to validate their safety and efficacy [260].

At various stages of clinical research, some compounds exhibit varying levels of progress and promise. In some cases, interest wanes after the first phase of trials, and often these studies are not continued. However, other compounds may show consistent results or promise through multiple stages, leading to further investigations and, in some cases, advancements toward approval. This disparity highlights the complexities and challenges faced in the clinical development of therapeutic agents, as well as the need for ongoing research to identify the most effective treatments.

A clinical trial investigating (NCT00462813) the effects of 3,3′-diindolylmethane supplementation on low-grade cervical cytological abnormalities revealed several key findings. Primarily, the study concluded that DIM supplementation did not significantly reduce the risk of developing CIN2+ or persistent HPV infection. While the supplement was generally well-tolerated, participants in the DIM group experienced a statistically significant increase in darkening of urine and reported a higher incidence of non-serious adverse events. Importantly, the trial was underpowered for its primary outcome, meaning it lacked sufficient participants to definitively assess DIM’s impact on CIN2+ histology, which limits the strength of conclusions regarding the primary outcome. Additionally, a discrepancy emerged between subjective and objective measurements: participants reported a statistically significant decrease in weight gain, yet objective weight measurements showed no significant difference between the DIM and placebo groups. This highlights the potential for variation between patient-reported outcomes and clinically measured data [261], NCT00462813 (Table 5).

There are numerous clinical trials investigating vincristine and its use in cancers such as hematological malignancies, neurological tumors, sarcomas, and retinoblastomas. Due to the vincristine toxicity, current research focuses on encapsulating vincristine in liposomes and studying its effectiveness primarily in hematological malignancies. A clinical trial investigated the efficacy and safety of vincristine sulfate liposome injection (VSLI) in relapsed or refractory Philadelphia chromosome-negative acute lymphoblastic leukemia. The study concluded that high-dose VSLI monotherapy resulted in meaningful clinical outcomes, including durable responses and the ability to bridge to hematopoietic cell transplantation, in a near-end-stage adult ALL population, with a manageable toxicity profile comparable to standard vincristine, despite the delivery of higher doses [262], NCT00495079. However, VSLI has received FDA accelerated approval for treating adults with relapsed or refractory ALL. It may also be beneficial in combination therapy for aggressive non-Hodgkin lymphoma [263], NCT00495079. Treatment with VSLI resulted in a substantial clinical advantage for adolescent young adult patients suffering from relapsed/refractory ALL [264], NCT00495079.

A study investigated the pharmacokinetics and safety of vincristine sulfate liposome injection (VSLI) with metastatic melanoma and impaired hepatic function. The researchers concluded that liver impairment did not significantly alter the pharmacokinetics of VSLI, possibly because the liposomal encapsulation shifts the drug’s clearance mechanism from solely liver-dependent processes to the reticuloendothelial system. Unfortunately, no significant clinical benefit was observed, and the median survival of the patients was only 25 days, likely due to the advanced stage of their cancer [265], NCT00506142 (Table 5).

A phase I clinical trial evaluated the safety, tolerability, and pharmacologic profile of vincristine sulfate liposome injection in combination with bendamustine and rituximab in patients with B-cell lymphomas. The study employed a dose-escalation design to determine the maximum tolerated dose (MTD) of VSLI when administered with standard doses of bendamustine and rituximab. The results demonstrated that the combination regimen was generally well-tolerated, with adverse events consistent with the known profiles of the individual agents. Importantly, the study successfully identified the MTD of VSLI in this combination setting, providing a clear basis for dosing in future trials. No unexpected toxicities were observed, and preliminary evidence suggested that the regimen could be administered safely to patients with relapsed or refractory B-cell lymphomas. These findings support the feasibility of combining VSLI with bendamustine and rituximab and provide a rationale for further investigation in larger phase II/III studies to assess efficacy and long-term safety outcomes NCT02257242 [266].

Another group of compounds showing promising therapeutic effects includes methanol and lycopene. A phase II study investigated the efficacy of a lycopene-rich tomato supplement in patients with androgen-independent prostate cancer. The trial included 46 asymptomatic patients with elevated prostate-specific antigen (PSA) levels despite hormonal therapy. Participants received a supplement containing 15 mg of lycopene twice daily and completed a questionnaire regarding their motivations for enrolling in an alternative medicine study. The results showed that only one patient (2%) had a confirmed PSA decline of 50% or more, indicating a minimal tumor response. Lycopene supplementation was generally well-tolerated, though one patient experienced grade 4 diarrhea and another died from a cancer-related hemorrhage. Other adverse events included mild to moderate diarrhea, nausea, abdominal distension, flatulence, vomiting, anorexia, and dyspepsia. Overall, the study concluded that the lycopene-rich tomato supplement did not demonstrate significant clinical efficacy in treating androgen-independent prostate cancer NCT00068731 [267].

A randomized, double-blind, placebo-controlled trial studied 30 mg/day lycopene supplementation for 21 days in 105 African American men undergoing prostate biopsy. Lycopene significantly increased plasma and prostate tissue levels (*p* < 0.01) but did not significantly affect oxidative stress biomarkers (8-oxo-dG in tissue or plasma malondialdehyde), though a trend toward reduced 8-oxo-dG was seen in men with BPH. The study demonstrates that short-term lycopene supplementation effectively increases systemic and prostate tissue lycopene levels but has limited impact on oxidative stress biomarkers in this population NCT00416390 [268].

The Phase II trial NCT01882985 investigated the combination of docetaxel chemotherapy with lycopene supplementation in patients with metastatic castration-resistant prostate cancer (mCRPC). Among the 13 enrolled patients, the PSA response rate (≥50% decline) was 76.9%, and the median time to PSA progression was 8 months, with a median overall survival of 35.1 months. The combination therapy was well-tolerated, and adverse events were consistent with those expected from docetaxel, with no new safety concerns identified. These findings suggest that lycopene supplementation may enhance the therapeutic efficacy of docetaxel without increasing toxicity and support further research into its role as an adjunctive treatment in mCRPC [NCT01882985].

In the study NCT00844792, supplementation with lycopene and fish oil was shown to induce significant changes in gene expression within prostate tissue. These alterations suggest potential biological mechanisms through which these micronutrients may influence the progression of prostate cancer, possibly by modulating pathways related to inflammation, cell cycle regulation, and apoptosis. This provides evidence that dietary components can have measurable molecular effects in the prostate NCT00844792 [269].

In the study NCT00402285, 84 men with low-risk prostate cancer were randomized to receive lycopene, fish oil, or placebo for three months. The analysis of gene expression in normal prostate tissue showed no significant changes in individual genes after supplementation with either lycopene or fish oil. However, pathway analysis revealed that both interventions modulated several important biological pathways, including those related to androgen and estrogen metabolism, arachidonic acid metabolism, and the Nrf2-mediated oxidative stress response. These findings suggest that although lycopene and fish oil supplementation did not alter specific genes significantly, they may influence molecular processes in prostate tissue that are relevant to prostate cancer prevention and progression NCT00402285 [270].

Methanol has garnered attention due to its potential in various biomedical applications. Randomized controlled trial, breast cancer patients receiving chemotherapy were assigned to either a topical menthol treatment or standard care for six weeks. Those who applied the 1% menthol solution to their hands and feet twice daily experienced a greater reduction in chemotherapy-induced peripheral neuropathy symptoms compared with the control group. The treatment was well tolerated, and the findings suggest that topical menthol can serve as an effective supportive therapy to alleviate neuropathic symptoms associated with chemotherapy in breast cancer patients NCT05429814 [271].

Observations from several studies indicate a complex landscape for clinical trials involving natural compounds. Specifically, some trials, such as those concerning apigenin, have been withdrawn for a variety of reasons, while others, like those investigating luteolin, remain in preparatory phases. An example of a withdrawn apigenin trial (NCT03139227) was a pilot clinical study designed to explore the potential health benefits of apigenin, a compound abundant in celery, for high-risk breast cancer patients. The primary objectives of this study were to assess the practicality of apigenin supplementation through a food-based approach, specifically using celery–banana, bread, and to establish its safety profile within this patient population. The intent was to provide crucial preliminary data for further research into apigenin’s potential therapeutic applications. In contrast, studies on luteolin are currently in the preparatory stage (NCT03288298), with one in vitro analysis designed to compare the pro-apoptotic effects of luteolin and its nano-formulation on tongue squamous cell carcinoma cells. This research aims to ascertain if nano-luteolin provides a more potent apoptotic response than standard luteolin. Therefore, while some natural compounds are being explored in clinical trials, the path from initial research to clinical application can be complex, with trials being withdrawn or remaining in preparatory phases for various reasons (Table 5).

Silibinin, a polyphenolic flavonoid, has demonstrated anti-neoplastic potential in preclinical cancer models, including prostate cancer. Phase I clinical trial aimed to evaluate the safety and tolerability of high-dose silybin-phytosome, a commercially available formulation of silibinin, in patients with advanced prostate cancer, and to determine a recommended phase II dose. This study concluded that high-dose silybin-phytosome is relatively well-tolerated in patients with advanced prostate cancer, with asymptomatic liver toxicity being the most commonly observed adverse event. Further research is warranted to explore the potential anti-tumor activity of silybin-phytosome, especially in combination with other therapeutic agents [272], NCT00487721. The second part of the study aimed to determine if silibinin, the active compound, would accumulate in prostate tissue and to assess its impact on relevant biomarkers. The findings of this study were that despite achieving high peak blood concentrations of silibinin, the compound did not accumulate in prostate tissue at notable levels after two weeks of therapy. This observation suggests that the transient nature of silibinin serum levels may not be sufficient for tissue penetration. While the study did not demonstrate significant changes in biomarkers, it highlights the need for further research to optimize silybinin delivery and explore its potential in prostate cancer treatment, possibly through longer treatment durations, sustained-release formulations, or combination therapies [273], NCT00487721 (Table 5).

Clinical trials phase I and II testing genistein with standard chemotherapy (FOLFOX/FOLFOX-bevacizumab) for metastatic colorectal cancer (mCRC). The results demonstrated that genistein was well-tolerated in combination with chemotherapy, with most adverse events attributed to the chemotherapy itself NCT01985763 [274]. Clinical trials also evaluate the impact of soy isoflavones, specifically genistein, on breast epithelial cell proliferation and expression of some genes. While the study did not demonstrate an overall favorable effect of soy isoflavones on breast epithelial cell proliferation, the observed increase in proliferation among premenopausal women raises concerns about potential pro-estrogenic effects. Additionally, the alterations in gene expression highlight the need for further research to understand the long-term implications of soy isoflavone consumption on breast tissue NCT00244933 [275]. Another clinical trial showed that genistein has the potential to reduce p-EGFR in bladder cancer tissue, indicating a possible therapeutic effect. However, the lack of a clear dose–response relationship and the observed trend towards reduced efficacy at higher doses suggest a potential bimodal effect [276], NCT00118040. Genistein-rich extract as a sole treatment did not significantly reduce PSA levels in most prostate cancer patients. However, a subset of patients in the active surveillance group showed potential benefits with PSA level decreases or stabilization [277], NCT00269555. In clinical trials, genistein is being tested in the form of genistein concentrated Polysaccharide (NCT00584532) and in combination with various compounds such as gemcitabine (NCT00244933), gemcitabine and erlotinib (NCT00376948) Il-2 (NCT022075112), decitabine (NCT01628471), and cholecalciferol (NCT01325311) (Table 5).

In the Phase 1b/2a trial, BIO 300 (genistein) was evaluated in combination with chemotherapy in 21 patients with advanced solid tumors. The treatment was well tolerated, with no dose-limiting toxicities observed. BIO 300 did not affect the pharmacokinetics of the chemotherapy agents, indicating no significant drug interactions. Preliminary efficacy was noted, with some patients achieving stable disease or partial responses. These findings support further investigation of BIO 300 in larger clinical trials NCT01628471 [278].

Currently, clinical trials are focused on studying the molecular mechanisms of resveratrol, including the Notch/WNT pathway, as well as its impact on the progression of gastrointestinal (NCT01476592) and colon cancer (NCT00256334, NCT00433576). Current clinical trials for pterostilbene are investigating the effects of megestrol acetate with or without pterostilbene in endometrial cancer patients undergoing hysterectomy (Table 5).

Clinical trials of natural compounds are not solely focused on investigating their anticancer properties, but also cardiovascular, neurological, endocrinological, and metabolic diseases. In conclusion, the study of natural compounds holds immense promise for developing novel therapies and treatments for critical diseases.

## 5. Conclusions and Future Directions

Despite the growing body of preclinical evidence highlighting the potential of natural compounds as modulators of the epigenetic landscape, the translation of these findings into clinical practice remains a formidable challenge. While numerous alkaloids, terpenes, and polyphenols have demonstrated the ability to influence epigenetic regulators such as DNA methyltransferases (DNMTs), histone deacetylases (HDACs), and histone acetyltransferases (HATs), the clinical utility of these compounds is often limited by fundamental pharmacokinetic and pharmacodynamic constraints. Many of these molecules display poor water solubility, chemical instability, and rapid metabolic degradation, which collectively result in low systemic bioavailability and restricted tissue distribution. Furthermore, their pleiotropic mechanisms of action, although beneficial from a multitarget therapeutic perspective, raise concerns about off-target effects and a lack of epigenetic specificity, potentially affecting both malignant and normal cells.

Efforts to overcome these limitations have focused on chemical derivatization, structural modification, and advanced delivery systems. Lipid-based carriers, nanoemulsions, solid lipid nanoparticles, and polymeric encapsulation have been explored to enhance solubility, protect compounds from metabolic degradation, and enable controlled release at the tumor site. Despite these advances, developing delivery strategies that also ensure compound stability remains a major challenge in clinical translation.

From an epigenetic standpoint, current research on natural compounds has primarily focused on DNA methylation, histone methylation and acetylation, and microRNA-mediated gene regulation—the canonical mechanisms underlying chromatin remodeling and transcriptional reprogramming. However, emerging studies suggest that natural compounds may also target less-studied layers of the epigenome, including histone lactylation, histone crotonylation, and N6-methyladenosine (m6A) RNA methylation. These novel modifications represent dynamic and reversible processes that expand the complexity of epigenetic regulation. Histone lactylation may connect metabolic reprogramming within the tumor microenvironment to epigenetic regulation. Likewise, m6A RNA methylation affects RNA stability and translation, introducing a new layer of gene expression control that can also be modulated by natural compounds. However, studies on these emerging mechanisms are still limited, and further comprehensive research is needed to clarify their biological roles and therapeutic potential.

Interestingly, despite their structural and chemical diversity, alkaloids, terpenes, and polyphenols seem to act on several common signaling pathways through their epigenetic mechanisms. These compounds influence gene expression networks that regulate key cellular processes, such as apoptosis, cell cycle arrest, DNA repair, migration, invasion, and angiogenesis. By reactivating tumor suppressors and suppressing oncogenes involved in cell proliferation and metastasis, natural epigenetic modulators can exert broad anticancer effects. This suggests that different natural compounds might work in combination to correct epigenetic changes and limit cancer progression.

In conclusion, natural compounds can influence the epigenome in diverse ways, offering promising strategies for cancer prevention and treatment. However, translating these preclinical findings into effective therapies requires a better understanding of their stability, bioavailability, target specificity, and delivery methods. Future studies should combine multi-omics approaches to capture the full range of epigenetic modifications—including newer marks such as histone lactylation, crotonylation, and RNA m6A methylation—and explore their effects on cellular function. Additionally, designing combination strategies with optimized formulations and targeted delivery systems may help overcome current pharmacological limitations. With such integrative approaches, natural compounds have the potential to move from experimental tools to clinically relevant epigenetic therapeutics.

## Figures and Tables

**Table 1:** Regulatory effects of alkaloids on miRNA expression.

Type of Natural Compound	Type of Cancer	miRNA	Cancer Association
Alkaloids			
3,3′-diindolylmethane	breast cancer	↑ miR-21	↓ Cdc25A ↓ cell cycle
sanguarine	pancreatic cancer	↓ miR-221	↑ PTEN, ↑ p27, ↑ p57, ↑ PUMA ↓ proliferation ↓ migration
		↑ miR-146	↓ EGFR, ↓ NF-κB ↓ invasive potential
evodiamine	colorectal cancer	↓ miR-429	↓ E-cadherin, ↓ Par3 ↓ proliferation ↓ migration ↑ caspase 3, ↑ PARP ↑ apoptosis
	ovarian cancer	↑ miR-152-3p	↓ CDK19 ↓ proliferation ↑ apoptosis
piperlongumine	lung cancer	↑ miR-34b-3p	↓ TGFBR1 ↓ cell growth ↓ migration ↓ invasion
	osteosarcoma pancreatic cancer	↓ miR-30d-5p ↓ miR-27a ↓ miR-17/20a	↑ SOCS3 ↓ JAK/STAT3 pathway ↓ proliferation, ↑ apoptosis ↓ metastasis ↓ c-Myc ↑ ZBTB10 ↑ ZBTB4
campthotecin	hepatocellular carcinoma	↓ miR-34b ↓ miR-222 ↑ miR-16	↓ cyclin D1, ↓ MMP2/9 ↑ p21, ↑ p27, ↓ TIMP1, ↓ E-cadherin
	breast cancer	↓ miR-125b	↑ Bak1, ↑ Mcl-1, ↑ p53
vincristine	leukemia	↓ miR-181a	↑ apoptosis

Legend: ↑ upregulation, ↓ downregulation.

**Table 2:** Regulatory effects of terpenoids on miRNA expression.

Type of Natural Compound	Type of Cancer	miRNA	Cancer Association
Terpenoids			
hinokitiol	breast cancer	↑ miR-494-3p	↑ *BMI1* ↑ mammosphere formation
betulinic acid	breast and colon cancer	↓ miR-27a	↓ Sp1/3/4 ↑ ZBT10A ↓ tumor volume and weight
	breast cancer	↓ miR-20a ↓ miR-106a/b	↓ Sp1/3/4 ↓ EZH2 ↑ ZBTB4
	pancreatic cancer	↓ miR-365	↑ BTG2 ↓ Il-6/AKT/Stat3
	hepatocellular carcinoma	↓ MALAT1 ↑ miR-22-3p	↑ apoptosis
oleanic acid	hepatocellular carcinoma	↑ miR-122	↓ migration ↓ invasion
	gastric cancer	↑ miR-98-5p	↓ Il-6
	chronic myeloid leukemia	↓ miR-18a-5p	↑ STK4 ↓ proliferation ↑ apoptosis
ursolic acid	breast cancer	↑ miR-149-5p	↓ MyD88 ↓ PI3K/Akt ↑ paclitaxel cell deadh
	breast cancer	↓ miR-499a-5p	↓ proliferation ↓ TCF/LEF ↓ Wnt/β-catenin ↑ SFRP4, ↑ DKK1 ↓ CSC markers (CD44, ALDH1, ABCC2, ABCG2)
	colorectal cancer	↑ miR-200a/c	↓ TGF-β1 ↓ Zeb1 ↓ growth ↓ aggressiveness
lycopene	prostate cancer	↑ miR-let-7f-1	↓ AKT2 ↓ cell growth ↑ apoptosis
astaxhantin	colon cancer	↑ miR-29a-3p ↑ miR-200a	↓ MMP2 ↓ Zeb1 ↓ metastasis
lutein	breast cancer	↑ miR-590-3p	↓ CASC9 ↓ proliferation

Legend: ↑ upregulation, ↓ downregulation.

**Table 3:** Summary of studies investigating the effects of selected polyphenols on epigenetic modifications and their associated molecular alterations and phenotypic outcomes in cancer cell models. Legend: (↑) indicates upregulation, and (↓) indicates downregulation.

Compound	Epigenetics Modification/ Changes in Level Enzymes Involved in Epigenetics Modifications	Molecular Changes	Cancer	Effect on Cancer Cells
**APIGENIN**	HDAC1-3 ↓ HAT ↑ DNMTs ↓ H3ac ↑ H4ac ↑	Bax ↑, Bid ↑, Bcl-2 ↓, p21/Waf1 ↑	breast cancer, prostate cancer	apoptosis ↑
**LUTEOLIN**	DNMT1 ↓ DNMT3A/B ↓ HDAC 1-3 ↓ HDAC 6,7 ↓	*NRF2* ↑	colorectal cancer	cell growth ↓ colony formation ↓
	TET1 ↑	NRF2 ↑, p53 ↑, Bax ↑, caspase 9 ↑, Bcl-2 ↓	colon cancer	apoptosis ↑
	DNMT1 ↓	Sp1 ↑, NFκB ↑, OPCML genes ↑	breast cancer	apoptosis ↑ cell growth ↓
	PRC2 ↓, EZH2 ↓, DU145 ↓ H3K27me3 ↓	CDH1 ↑, SLIT2 ↑, TIMP3 ↑	prostate cancer	proliferation ↓ invasiveness ↓
	p300 ↓ H3K9ac ↓, H3K14ac ↓, H4ac ↓	Ki67 ↓, Il-6 ↓, ADORA1 ↓, TENMT1 ↓	oral cancer	tumor progression ↓
	H3ac ↑	Fas ↑, FasL ↑, caspase 8 ↑	leukemia	apoptosis ↑
	H3K56ac ↑, H3K27ac ↑	AKT/mTOR ↓, MMP9 ↓	breast cancer	proliferation ↓, metastasis ↓
**CHRYSIN**	DNMT3A/3B ↓, HDACs ↓, HAT1 ↓, EZH2 ↓, PRMT8 ↓, AURK ↓ SEDT2 ↑, ESCO2 ↑, CITA ↑	*APC* ↓, *BRCA1* ↓, *CDH1* ↓, *PTEN* ↓, *GSTP1* ↓, *FHIT* ↓, *CDKN2A* ↓, *DAPK1* ↓, *MGMT* ↓, *MLH1* ↓, *RARβ* ↓, *RASSSF1* ↓, *SOCS1* ↓, *TIMP3* ↓, *VHL* ↓, *WIF1* ↓	breast cancer	proliferation ↓, metastasis ↓
	H3K9me3 ↓, H3K27me ↓, H3K36me ↓, H3K79me ↓, H3K4me ↓, H3K4me ↓, H3ac ↓, H4ac ↓	-	breast cancer	proliferation ↓, metastasis ↓
	TET1 ↑ 5hmC ↑	-	gastric cancer	apoptosis ↑, migration ↓, invasion ↓, growth ↓
	DNMT1 ↓ global hyperacetylation	p21 ↑, CTPS ↓	breast cancer	growth ↓
	m6A	ENO1 ↓, WNT/β-catenin ↓	hepatocellular carcinoma	EMT ↓
**KAEMPFEROL**	DNA hypomethylation ↑	WNR genes ↑	bladder cancer	DNA repair ↓
	DNA hypermethylation ↑	RCF genes ↓	bladder cancer	growth ↓
	DNMT1 ↓, DNMT3A/3B ↓	PI3K/AKT ↓	bladder cancer	tumor progression ↓
	HDAC 2,4,7,8 ↓ H3ac ↑	-	hepatoma, colon cancer	viability ↓ proliferation ↓
	G9a ↓ H3K9me2 ↓	-	gastric cancer	autophagy ↑
**MYCERTIN**	KDM4A,B,C ↓ H3K9me3 ↓	-	prostate cancer	proliferation ↓
**FISETIN**	KDM4A ↑ H3K36me ↓	RFXAP ↑	pancreatic carinoma	proliferation ↓ gemcitabine sensitivity
	TET1 ↓	CCNY ↓, CDK16 ↓	renal cancer	Cell cycle progression ↓
	m6A methylation	ZC3H13 ↓, PHF ↓, γH2AX ↓, RAD51 ↓	pancreatic ductal carcinoma	DNA repair ↓ sensitive to chemiotherapy
**RHAMNETIN**	HDAC2 ↓	-	ovarian cancer	proliferation ↓ apoptosis ↑
**HESPEDRIN**	global hypomethylation	LINE, ALU2 ↑	leukemia	chemopreventive potential apoptosis ↑ angiogenesis ↓
**SIBILIBIN**	PRC2 ↓, EZH2 ↓, SUZ11 ↓, EED ↓, DNMT ↑, HDAC1,2 ↓	pAKT(S473) ↓ pEZH2 ↓	prostate cancer	EMT ↓ stem cell properties ↓
	H3ac ↑, H4ac ↑	-	hepatocellular carcinoma	growth ↓
	H3K4me3 ↓ H3ac ↓	KRAS ↓	bladder cancer	proliferation ↓ invasion ↓
**GENISTEIN**	DNMT3B ↓	CDH5 ↓	neuroblastoma	growth ↓
	DNMT1 ↓	MBD2 ↓	prostate cancer	proliferation ↓
	H3ac ↑, H4ac ↑, H3k4me2 ↑, H3k4me3 ↑	BTG3 ↑	prostate cancer	proliferation ↓
	p300 ↑, PCAF ↑, CREBBP ↑, HAT1 ↑ H3ac ↑, H4ac ↑, H3K4me2 ↑	p16 ↑,p21 ↑	prostate cancer	growth ↓
	DNMT1/3A/3B ↓, HDAC1,5,6 ↓ SEDT5-7 ↑, CITA ↑, ESCO2 ↑	*APC* ↓, *DAPK1* ↓, *FHIT* ↓, *PTEN* ↓, *GSTP* ↓, *RARβ* ↓, *RASSSF1* ↓, *CDH1* ↓, *MLH1* ↓, *SOCS1* ↓, *TIMP3* ↓, *CDH13* ↓, *MGMT* ↓, *VHL* ↓	cervical cancer	migration ↓ angiogenesis ↓ proliferation ↓ apotosis ↑
	H3ac ↓	procaspase 9 ↑, cyclin D1 ↓	breast cancer	proliferation ↓ apoptosis ↑
	H3ac ↓	DKK1 ↑, WNT/β-catenin ↓	colon cancer	proliferation ↓ cell cycle arrested
**DAIDZEIN**	methylation	*BRCA1* ↓, *GSTP1* ↓, *EPHB2* ↓	prostate cancer	cell cycle arrested
	hypermethylation	*BRCA1* ↓, *BRCA2* ↓	breast cancer	Cancer development
	p300 ↑, EZH2 ↓ H3K9me3 ↓, H3K27me ↓ H4K8ac ↑, H3K4ac ↑	p300 ↑, EZH2 ↓ SCR ↑, BRCA1 ↑, Erα/β ↑	breast cancer	proliferation ↓ migration ↓ invasion ↓
**DELPHINIDIN**	p300 ↓, EZH2 ↓ DNA methylation	NRF-2 ↓, HO-1 ↑	skin cancer	prevents neoplastic transformation
**RESVERATROL**	hypermethylation	Notch ↓, Wnt ↓, Hedgehog ↓, TGF-β ↓, MAPK ↓, AKT ↓, GLI2 ↓, WNT4 ↓ Bcl2 ↓, Epcam ↓, CCND1 ↓, CYR61 ↓	breast cancer	proliferation ↓ metastasis ↓ apoptosis ↑
	H3K27me3 ↑, H3K17ac ↓, H3K9ac ↓	-	breast cancer	proliferation ↓ metastasis ↓ apoptosis ↑
	hypomethylation hypermethylation	IGFR2R ↑, TP53 ↑, FOXO3 ↑,SOX17 ↑, SLIT3 ↑, CDO1 ↑ AURKA ↓, CCNB1 ↓, HK2 ↓	breast cancer	progression ↓
	PRTM5 ↓, EZH2 ↓ H4R3me2 ↓, H3K27me3 ↓	-	breast cancer	proliferation ↓
	HDAC ↓, KAT2A ↑, KAT3B ↑ H3K9ac ↑, H3K27ac ↑	BRCA1 ↑, p53 ↑, p21 ↑	breast cancer	proliferation ↓
	HDAC1 ↓ H3 hypoacetylation	-	hepatoma	proliferation ↓
**RESVERATROL+PTEROSTILBENE**	SIRT1 ↓	yH2AX ↓, hTERT ↓	breast cancer	Cell cycle arrested apoptosis ↑
**PTEROSTILBENE**	DNMT3B ↑ hypermethylation	*OCT1* ↓, *PRKCA* ↓, *DANT2* ↓, *TNNT2* ↓	breast cancer	adhesion ↓ growth ↓ apoptosis ↑
	MTA1 ↓, HDAC1 ↓, HAT ↑	PTEN acetylation, pAKT ↓	hepatocellular carcinoma	adhesion ↓ growth ↓ apoptosis ↑ invasion ↓
**GALLIC ACID**	DNMT3B ↓ DNMT1 ↓	CCNE2/3 ↑, CDKN1A ↑, CCNB1 ↑	lung and oral cancer	growth ↓
**ELLAGIC ACID**	H3K27me3 ↓ H4R3me2 ↓	Ki-67 ↓	prostate cancer	proliferation ↓

**Table 4:** Regulatory effects of polyphenols on miRNA expression.

Type of Natural Compound	Type of Cancer	miRNA	Cancer Association
Polyphenols			
apigenin	breast cancer	↓ miR-21 ↑ miR-200b	↑ apoptosis
	colon cancer	↑ miR-215-5p	↓ E2F1 ↓ E2F3 ↓ cell cycle
	glioma	↑ miR-16	↓ Bcl2 ↓ NF-κB ↓ MMP-9 ↓ invasive potential ↑ apoptosis
	liver cancer	↓ miR-101	↑ NRF2 ↑ apoptosis
	glioma	↑ miR-103a-3p	↓ NEDD9 ↓ FAK/AKT ↓ cell proliferation ↓ migration ↓ invasion ↓ EMT
luteolin	pancreatic cancer	↓ miR-301-3p	↑ caspase 8 ↑ TRAIL-induced apoptosis
	lung cancer	↑ miR-106a-5p	↓ TWIST1 ↓ *MMP2*
	gastric cancer	↑ miR-34	↓ HK1 ↓ tumor growth
chrysin	gastric cancer	↑ let-7 ↑ miR-9 ↑ miR-22 ↑ miR-34 ↑ miR-126 ↓ miR-18a ↓ miR-21 ↓ miR-221	↓ proliferation ↓ differentiation ↓ migration ↓ invasion ↓ angiogenesis ↑ apotosis
kaempferol	colon cancer	↑ miR-339-5p	↓ hnRNPA1 ↓ PTBP1 ↓ PKM2 ↑PKM1 ↓ cell proliferation ↓ glycolisis
rhamnetin	breast cancer	↑ miR-34a	↑ caspase 3 ↑ caspase 9 ↑ p53 ↓ Notch
	non-small-cell lung cancer	↑ miR-34a	↓ Notch sestitivity to radiotherapy ↑ apoptosis ↓ cell proliferation ↓ EMT
	hepatocellular carcinoma	↑ miR-148a	↓ PXR ↓ CYP3A4 ↓ P-GP ↓ Sirt1 ↑ p53 acetylation sensitivity to sorafenib
hesperidin	hepatocellular carcinoma	↓ miR-221 ↑ miR-34a	↓ FGF-2 ↑ Nrf2 ↑ Bcl-2 ↑ caspase 3 ↓ angiogenesis ↑ apoptosis
	non-small cell lung cancer	↑ miR-132	↓ ZEB2 ↓ invasion ↓ metastasis ↑ apoptosis
	colon cancer	↑ miR-34a	↑ p53 ↓ EGFR ↓ PD-L1
	breast cancer	↑ miR-34a ↑ miR-16 ↓ miR-21	↓ Bcl-2 ↑ apoptosis
naringenin	leukemia	↑ miR-34a	↓ Bcl-2 ↓ XIST ↓ HDAC1 ↑ caspase 3
silibinin	breast cancer	↓ miR-21 ↓ miR-155	↑ caspase 9 ↑ p53 ↑ BID
	breast cancer	↓ miR-21	↑ *PDCD4*, ↑ *PTEN*, ↑ *FASL*, ↑ *CASP*, ↑ p53 ↑ apoptosis ↓ proliferation ↓ tumor growth
	colorectal cancer	↑ miR-34a ↓ miR-221 ↓ miR-222	↑ p53, ↑ BAX, ↑ caspase 9, ↑ caspase 3, ↑ caspase 8, ↓ Bcl-2, ↑ apoptosis
	non-small cell lung cancer	↓ miR-21 ↑ miR-200c	↓ SNAIL1 ↓ ZEB1 ↓ ZEB2 ↓ N-cadherin ↓ EMT ↓ migration erlotinib-sensitive
	breast cancer	↓ miR-20b	↑ BIM ↑ PTEN ↑ caspase 9
	breast cancer	↑ miR-133a	↓ EGFR, ↓ PIK3C2A ↓ tumor growth
genistein	lung cancer	↑ miR-27a	↓ MET ↓ proliferation
daidzein	glioblastoma	↓ miR-137	↓ Akt, NF-κB, VEGF, b-FGF, EGFR, MMP-9, and MMP-2, ↓ cell survival ↓ angiogenesis
delphinidin	breast cancer	↑ miR-34a	↓ HOTAIR genes ↓ cancer progression
	colorectal cancer	↑ miR-204-3p	↓ αV/β3-integrin/FAK ↓ (FAK)/Src/paxillin ↓ migration ↓ invasion
resveratrol	bladder cancer	↓ miR-21	↑ apoptosis ↓ Bcl-2 ↑ caspase 3 ↓ AKT activity
	leukemia	↑ miR-150	↓ MDM2 ↓ RUNX3 ↓ RB ↑ apoptosis
pterostilbene	prostate cancer	↓ miR-20a	↑ MICA/B, ↓ TGF-β1 ↑ cytotoxicity
	prostate cancer	↓ miR-17 ↓ miR-20a ↓ miR-106a/b	↑ apoptosis
	hepatocellular carcinoma	↓ miR-19a	↑ PTEN ↓ PI3K/Akt ↓ cell viability ↓ cell invasion ↑ apotosis ↑ cell arrested
	endometrial cancer	↑ miR-663b	↓ BCL2L14 ↑ apotosis
piceatannol	osteocarcinoma	↓ miR-21	↑ PTEN ↓ PI3K/Akt ↓ growth ↑ apotosis
	colorectal cancer	↑ miR-129	↑ Bax ↓ Bcl-2 ↑ caspase 3 ↑ apotosis
	pancreatic cancer	↑ miR-125b	↑ Bax ↓ Bcl-2 ↑ caspase 3 ↑ apotosis
	melanoma	↑ miR-181a	↑ Bax ↓ Bcl-2 ↑ caspase 3 ↑ apotosis
gallic acid	chondrosarcoma	↑ miR-518b	↑ Bax ↓ Bcl-2 ↑ caspase 3 ↑ caspase 3 ↑ apotosis
	colorectal cancer	↑ miR-1247-3p	↓ integrin αV/β3 ↓ paxillin activation
	liver cancer	↑ miR-1247-3p	↓ tumor growth ↓ integrin/FAK axis
	glioblastoma	↑ miR-17-3p ↑ miR-21-5p ↑ miR-421-5	↓ antioxidant activity ↓ tumor growth ↓ repair cellular damage

Legend: ↑ upregulation, ↓ downregulation.

**Table 5:** Clinical trials of natural compounds in cancer treatment.

Natural Compounds	Type of Cancer	Clinical Trials Number	Phase	Status	Results
**Alkaloids**					
**3,3′-diindolylmethane**	cervical cancer	NCT00462813	III	completed	✓ DIM supplementation did not significantly reduce the risk of CIN2+ or persistent HPV infection with patients with low-grade cervical cytological abnormalities; ✓ Generally well-tolerated, but increased urine darkening and more minor side effects were reported; ✓ Underpowered for the primary outcome, limiting definitive conclusions; ✓ Participants reported less weight gain, but measured weight showed no difference.
**vincristine**	acute lymphoblastic leukemia	NCT00495079	II	completed	✓ High-dose vincristine sulfate liposome injection (VSLI) produced durable clinical responses in relapsed/refractory Philadelphia chromosome-negative ALL. ✓ Side effects were similar to standard vincristine, even at higher doses. ✓ VSLI allowed patients to proceed to stem cell transplantation and benefited adolescents and young adults. ✓ FDA accelerated approval for adults with relapsed/refractory ALL; may also be useful in combination therapy for aggressive non-Hodgkin lymphoma.
	malignant uveal melanoma	NCT00506142	II	completed	✓ Liver impairment did not significantly affect VSLI metabolism. ✓ Liposomal formulation may shift clearance from the liver to the reticuloendothelial system. ✓ No significant clinical benefit observed in metastatic melanoma. ✓ Median survival was only 25 days, reflecting advanced disease stage.
	B-cell lymphoma	NCT02257242	I	completed	✓ Phase I trial evaluating VSLI with bendamustine and rituximab in patients with B-cell lymphomas. ✓ Determine the maximum tolerated dose (MTD) of VSLI in combination therapy. ✓ The combination was well-tolerated, with side effects consistent with known profiles of the drugs. ✓ Safe dosing level of VSLI identified for future studies. ✓ No unexpected or severe toxicities observed. ✓ The regimen is feasible and safe, providing a basis for further clinical trials to evaluate efficacy.
**Terpenoids**					
**lycopene**	prostate cancer	NCT00006078	I	completed	No results posted
		NCT00068731	II		✓ Patients: 46 men with androgen-independent prostate cancer. ✓ Intervention: Lycopene-rich tomato supplement, 15 mg twice daily. ✓ Efficacy: Only 1 patient (2%) showed a ≥50% PSA decline. ✓ Generally well-tolerated; most side effects were mild (diarrhea, nausea, abdominal distension, flatulence, vomiting, anorexia, dyspepsia). ✓ One patient had grade 4 diarrhea, another died from cancer-related hemorrhage. ✓ No significant clinical benefit observed from the supplement.
		NCT00416390	not applicable		✓ Plasma lycopene increased ~2-fold in the lycopene group (*p* < 0.0001). ✓ Lycopene levels in prostate tissue were significantly higher than in placebo (*p* = 0.005). ✓ No significant reduction in tissue 8-oxo-dG or plasma malondialdehyde; slight trend toward lower 8-oxo-dG in men with BPH. ✓ Well-tolerated, with very few mild side effects. ✓ Lycopene effectively increases plasma and tissue levels but does not substantially reduce oxidative stress.
		NCT00093561	I		No results posted
		NCT01443026	II		No results have been fully posted.
		NCT00042731	not applicable		No results posted
		NCT00178113	I		No results posted
		NCT00450749	II		No results posted
		NCT01882985	II		✓ PSA response: ≥50% decline in 76.9% of patients ✓ Median time to PSA progression: 8 months ✓ Median overall survival: 35.1 months ✓ Treatment well-tolerated, adverse events consistent with docetaxel, no new safety concerns ✓ Lycopene may enhance the efficacy of docetaxel without increasing toxicity, supporting further investigation
		NCT00416325	I		No results posted
		NCT01105338	II, III		No results posted
		NCT00844792	II		No results posted
		NCT00402285	not applicable		✓ No single genes showed significant differential expression after supplementation with lycopene or fish oil compared to placebo. ✓ Lycopene and fish oil supplementation modulated pathways related to androgen/estrogen metabolism and arachidonic acid metabolism. ✓ Both supplements also influenced the Nrf2-mediated oxidative stress response pathway. ✓ Although no individual gene changes were statistically significant, lycopene and fish oil supplementation appeared to affect key molecular pathways in prostate tissue, suggesting possible biological activity relevant to prostate cancer prevention.
		NCT00744549	II		No results posted
**menthol**	breast cancer	NCT05429814	not applicable	completed	✓ Breast cancer patients undergoing chemotherapy who received a 1% topical menthol treatment applied twice daily to the hands and feet for six weeks showed greater improvement in chemotherapy-induced peripheral neuropathy symptoms compared with those receiving standard care. ✓ The treatment was well tolerated, with no major adverse effects reported. ✓ The study concluded that topical menthol may be an effective supportive therapy for reducing neuropathic symptoms in breast cancer patients receiving chemotherapy.
**Polyphenols**					
**apigenin**	breast cancer	NCT03139227	not applicable	withdrawn	No results posted
**luteolin**	tongue squamous cell carcinoma	NCT03288298	early phase I	not yet recruiting	No results posted
**silibinin**	prostate cancer	NCT00487721	II	completed	✓ High-dose silybin-phytosome was generally well tolerated in patients with advanced prostate cancer. ✓ The most common adverse event observed was asymptomatic liver toxicity. ✓ Despite achieving high blood concentrations, silibinin did not accumulate in prostate tissue after two weeks of treatment. ✓ No significant changes were observed in prostate cancer biomarkers. ✓ The findings suggest that short-term silibinin therapy may not ensure sufficient tissue penetration. ✓ Further studies are needed to optimize silibinin delivery and evaluate its potential anti-tumor effects, particularly with longer treatments or in combination with other therapies.
**genistein**	colorectal cancer	NCT01985763	I, II	completed	✓ Phase I/II pilot study tested genistein with FOLFOX or FOLFOX–Bevacizumab in metastatic colorectal cancer; genistein was well tolerated, with mild adverse events such as headaches, nausea, and hot flashes, and did not increase chemotherapy toxicity. ✓ Best overall response rate 61.5%; median progression-free survival 11.5 months. ✓ Genistein combined with standard chemotherapy is safe, tolerable, and shows promising efficacy, supporting further research.
	breast cancer	NCT00290758 NCT00244933	II II	completed	✓ Soy isoflavone supplementation did not reduce breast epithelial proliferation in high-risk women. ✓ In premenopausal women, breast epithelial proliferation slightly increased. ✓ Supplementation induced changes in the expression of genes related to proliferation, apoptosis, and estrogen effects. ✓ No significant changes were observed in cytologic atypia or nipple aspirate fluid biomarkers. ✓ Findings suggest soy isoflavones may not be effective for breast cancer prevention and require further study regarding safety and long-term effects.
	squamous cell carcinoma of the head and neck	NCT02075112	I	completed	✓ The combination of gemcitabine hydrochloride and genistein in 19 women with stage IV breast cancer to evaluate response rate, survival, time to progression, toxic effects, and correlation with plasma genistein levels. ✓ Detailed results have not been published or made publicly available. ✓ The efficacy and safety of this combination remain unclear. ✓ Further research is needed to assess the potential benefits and risks of combining gemcitabine with genistein in advanced breast cancer.
	melanoma and kidney cancer	NCT00276835	early phase I	completed	No results posted
	bladder cancer	NCT00118040	I	completed	✓ In this Phase 2 randomized, placebo-controlled trial, 59 patients with urothelial bladder cancer received daily oral genistein (300 or 600 mg/day) for 14–21 days before surgery, and the treatment was well tolerated, with most toxicities being mild to moderate and not attributed to the drug. ✓ Biomarker analysis aimed to assess the impact of genistein on molecular pathways in bladder epithelial tissue, though specific outcomes were not detailed. ✓ These findings suggest genistein is safe for short-term use in bladder cancer patients and may influence cancer-related molecular pathways, warranting further research.
	prostate cancer	NCT00269555	not applicable	completed	✓ The combination of a single high dose of cholecalciferol (200,000 IU) and daily genistein (600 mg) was well tolerated by patients with early-stage prostate cancer undergoing prostatectomy. ✓ The study aimed to assess the impact of this combination on prostate tissue concentrations of calcitriol and related biomarkers. However, specific outcomes regarding biomarker changes were not detailed in the available information. ✓ This was a randomized, placebo-controlled trial, indicating a rigorous approach to evaluating the interventions.
		NCT00584532	II, III		No results posted
		NCT01325311	II		No results posted
		NCT00078923	II		No results posted
	non-small cell lung cancer	NCT01628471 NCT02567799	I, II I, II	completed	✓ The Phase 1b/2a trial evaluated BIO 300 (genistein) combined with chemotherapy in 21 patients with advanced solid tumors, and the treatment was well tolerated with no dose-limiting toxicities. ✓ The drug did not affect chemotherapy pharmacokinetics, indicating no significant interactions. ✓ Preliminary efficacy was observed, with some patients achieving stable disease or partial responses. ✓ The findings support further investigation of BIO 300 in larger trials. No results posted
	pancreatic cancer	NCT00376948	II	completed	No results have been fully posted
**resveratrol**	gastrointestinal tumors	NCT01476592	not applicable	completed	No results posted
	colon cancer	NCT00256334 NCT00433576	I I	completed	No results posted ✓ No effect on Wnt gene expression in tumor tissue. ✓ Significant reduction of Wnt activity in normal colorectal mucosa. ✓ 80 g grape powder/day showed the strongest effect. ✓ Effect confirmed in vitro in normal colon cells. ✓ Suggests potential chemopreventive effect in healthy tissue only.
**pterostilbene**	endometrial cancer	NCT03671811	II	active, not recruting	No results posted

## Data Availability

No new data were created or analyzed in this study. Data sharing is not applicable to this article.
